# Multiple and diversified transposon lineages contribute to early and recent bivalve genome evolution

**DOI:** 10.1186/s12915-023-01632-z

**Published:** 2023-06-26

**Authors:** Jacopo Martelossi, Filippo Nicolini, Simone Subacchi, Daniela Pasquale, Fabrizio Ghiselli, Andrea Luchetti

**Affiliations:** 1 Department of Biological Geological and Environmental Science, University of Bologna, Via Selmi 3, 40126 Bologna, Italy; 2 Fano Marine Center, Department of Biological, Geological and Environmental Sciences, University of Bologna, Viale Adriatico 1/N, 61032 Fano, Italy

**Keywords:** Mollusca, Evolutionary genomics, TE classification, Bivalvia, Comparative genomics, SINE, TE evolution, TE annotation

## Abstract

**Background:**

Transposable elements (TEs) can represent one of the major sources of genomic variation across eukaryotes, providing novel raw materials for species diversification and innovation. While considerable effort has been made to study their evolutionary dynamics across multiple animal clades, molluscs represent a substantially understudied phylum. Here, we take advantage of the recent increase in mollusc genomic resources and adopt an automated TE annotation pipeline combined with a phylogenetic tree-based classification, as well as extensive manual curation efforts, to characterize TE repertories across 27 bivalve genomes with a particular emphasis on DDE/D class II elements, long interspersed nuclear elements (LINEs), and their evolutionary dynamics.

**Results:**

We found class I elements as highly dominant in bivalve genomes, with LINE elements, despite less represented in terms of copy number per genome, being the most common retroposon group covering up to 10% of their genome. We mined 86,488 reverse transcriptases (RVT) containing LINE coming from 12 clades distributed across all known superfamilies and 14,275 class II DDE/D-containing transposons coming from 16 distinct superfamilies. We uncovered a previously underestimated rich and diverse bivalve ancestral transposon complement that could be traced back to their most recent common ancestor that lived ~ 500 Mya. Moreover, we identified multiple instances of lineage-specific emergence and loss of different LINEs and DDE/D lineages with the interesting cases of CR1- Zenon, Proto2, RTE-X, and Academ elements that underwent a bivalve-specific amplification likely associated with their diversification. Finally, we found that this LINE diversity is maintained in extant species by an equally diverse set of long-living and potentially active elements, as suggested by their evolutionary history and transcription profiles in both male and female gonads.

**Conclusions:**

We found that bivalves host an exceptional diversity of transposons compared to other molluscs. Their LINE complement could mainly follow a “stealth drivers” model of evolution where multiple and diversified families are able to survive and co-exist for a long period of time in the host genome, potentially shaping both recent and early phases of bivalve genome evolution and diversification. Overall, we provide not only the first comparative study of TE evolutionary dynamics in a large but understudied phylum such as Mollusca, but also a reference library for ORF-containing class II DDE/D and LINE elements, which represents an important genomic resource for their identification and characterization in novel genomes.

**Supplementary Information:**

The online version contains supplementary material available at 10.1186/s12915-023-01632-z.

## Background

Transposable elements (TEs) are selfish genetic elements that replicate independently from the replication of the host genome [1, 2]. They are widespread and ubiquitous across all branches of the eukaryotic tree of life and, although showing a remarkable sequence diversity across organisms, the conservation of common catalytic domains responsible for their replication suggests that their emergence could be traced back to the eukaryotic most recent common ancestor or even predate it [3].

TE classification is not straightforward, although many efforts have been undertaken to try to reconcile their diversity in a systematic framework. Two main classes are generally recognized: class I, which includes all TEs replicating via RNA intermediates, and class II, which embodies TEs moving via DNA intermediates [4]. This latest distinction still represents the only unambiguous classification of TEs. Conversely, the within-class diversity is much more complicated to analyze, since it can be performed both with mechanistic and homology-based criteria [5]. For example, considering the way TEs replicate and reintegrate, all class I elements use a “copy-and-paste” mechanism, while class II exhibits several models: the classical “cut-and-paste,” or the “peel-and-paste” (also known as rolling-circle replication) or even the “self-synthesizing” model (reviewed in [5]). The current classification scheme, which is also implemented in the main TE database, Repbase [6], is based on homology and structural similarities [7]. Class I elements mainly include long terminal repeat (LTR) elements and long interspersed nuclear elements (LINEs, also indicated as non-LTR elements) which encode for a reverse transcriptase (RT), an endonuclease (EN), and other domains used to reintegrate in the host genome. Class II elements, on the other hand, include terminal inverted repeat (TIR) elements, Helitrons, and Mavericks (also known as *Polintons*). In addition, both classes include non-autonomous elements (short interspersed nuclear elements, SINEs, and miniature inverted-repeats transposable elements, MITEs), TEs usually with a smaller size, which do not code for the enzymes necessary for replication/reintegration but parasitize those encoded by their autonomous counterparts [7]. Besides this commonly accepted scheme, further classification efforts are less clear. Generally speaking, when taking into consideration coding TEs, the clustering pattern after a phylogenetic analysis of their ORF(s) is taken as an indication of clades that should be considered possible families, groups of elements, or clades [5].

Although a common approach, the phylogenetic framework has limitations in this context both because of the sometimes unclear homology of TE ORFs and the genomic turnover of paralogous TE lineages blurring the phylogenetic signal [8].

The same replicative dynamics of TEs may impact their phylogenetic clustering: in fact, based on studies on mutation distribution on non-autonomous class I Alu sequences in the human genome, two distinct models have been formulated to explain how TEs replicate [9]. The first model, named “master gene model,” implies that one or few copies give origin to all other copies in the genome producing new, so-called families each time a master copy mutates. This way, new families are generated in different timeframes. On the contrary, in the other model, termed “transposons model,” each new copy can produce other copies with the outcome of getting several families produced nearly at the same time.

The rate at which TEs replicate can be a function of several different factors, including the ability of the host genome to limit their uncontrolled proliferation. In particular, the successful invasion of a genome by TEs can be dependent on a complex interplay among TE features, host genome biology, repression mechanisms interfering with TE functionality, and the extent of selective pressures on the outcome of TE insertions [10]. Despite this, some TE lineages managed to reach very high copy numbers in the host genomes, apparently escaping such controlling mechanisms. A suitable model to explain these dynamics has been formulated on the well-studied human SINE family Alu and on their autonomous counterparts L1 LINEs. These elements show several subfamilies that evolved following a master gene model in different hominid lineages during the last few million years. However, their origin seems to predate their species-specific expansions by far, with little or no transposition for tens of million years. Han et al. [11] hypothesized that the species-specific rise to a high copy number of some subfamilies could be due to some “stealth drivers,” i.e., Alu and L1 copies with a very low activity which allowed them to survive, undetected, in different host lineages, and that suddenly underwent a massive replication wave in specific conditions in given hosts.

Despite being extensively analyzed among vertebrates and arthropod genomes, TEs are surprisingly understudied in the phylum Mollusca, a large and diverse group of metazoans with many ecologically and economically important species. To date, TE studies in molluscs are limited to the characterization of one or a few elements [12–22] or to the whole mobilome, i.e., the full complement of TEs in the genome, but in a few species [23–25]. A direct consequence of the lack of genome-scale analyses of TE content in mollusc genomes is that public repositories and databases only harbor scarce information about them, making de novo assembled genome annotations less reliable [26, 27]. Therefore, besides the importance of analyzing the TE content and their relationships with host genomes in molluscs, it is also crucial for future genomic studies to get more detailed and wider TE libraries available.

In the present work, we leveraged the mollusc genome resources currently available in public databases, with a particular focus on bivalves, and carried out an extensive study of the full mobilome. An in-depth analysis of class II DDE/D-related transposons and LINEs allows us to deeply characterize an ancestral TE complement and its following expansion and contractions coupled with bivalve evolutionary history. Moreover, we manually curated a representative set of LINEs and DDE/D families that correspond to potentially recently active elements. The curated LINE library was finally used to reconstruct LINE evolutionary histories and assess their potential activity in male and female gonads of 5 species distributed across 4 different bivalve orders. The DDE/D and LINE manually curated library produced in this work could represent an important future resource for the bivalve genomic community to improve TE annotation in novel genomes.

## Results

### Overall TE content across molluscs using automatically generated TE sequence libraries

**Fig. 1:**
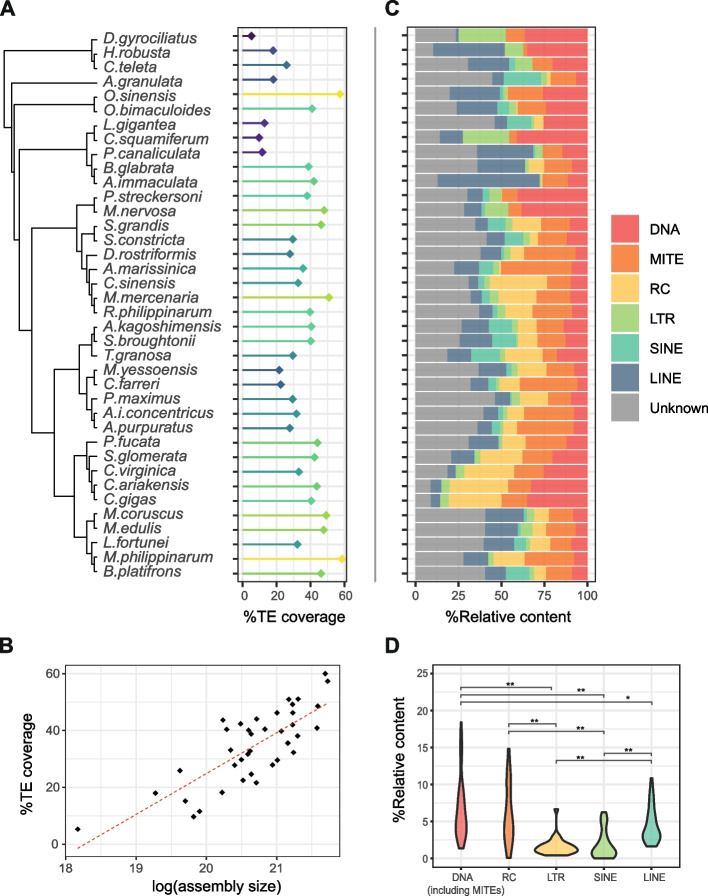
Transposable element content across molluscs. TE annotation results from automatically generated TE sequence libraries (see the “Genomic resources and phylogeny construction” section). **A** Phylogeny of the 39 analyzed genomes as retrieved from the literature and their overall transposable elements (TEs) content. **B** Correlation between TE coverage and assembly size as a proxy of genome size. **C** Relative contribution of different TE classes to the total TE content across molluscs. **D** Genome occupancy of each TE class in the 27 analyzed bivalves. Significant comparisons are highlighted by asterisks (pairwise Wilcoxon rank test with Bonferroni correction; *p < 0.05, **p < 0.01). A specular box plot considering all analyzed species, including other molluscan classes and annelids, is presented in Additional file 6: Fig. S3

To analyze the mollusc mobilome, we compiled a dataset of 39 molluscan genomes representative of their major groups (Additional file 1: Table S1). Among these, 27 belong to bivalve species and represent eight different orders: Unionida, Adepedonta, Myida, Venerida, Arcida, Pectinida, Ostreida, and Mytilida. As a first step, we implemented an automatic TE annotation pipeline (see the “Mining and annotation of interspersed repeats” section; Additional file 2: Fig. S1) which identified a variable number of consensus sequences, ranging from 92 elements in the annelid *Dinophilus gyrociliatus* to the 3736 elements in the Mytilida *Modiolus philippinarum* (Additional file 3: Table S2). When annotating each genome with the corresponding species-specific library, as expected from an understudied phylum such as molluscs, “unknown” elements represent a considerable proportion of the annotated repeats (mean = 10.41%; Fig. 1A; Additional file 4: Table S3), especially in poorly studied taxa such as *Solen grandis* (16.12%) and *Mytilus coruscus* (20.07%). Segmental duplications and recently duplicated gene families could be one of the major sources of unclassified TE consensus; however, we tried to reduce their impact by removing gene and gene fragments from the repeat library and by requiring at least 5 positive blast hits (with at least 70% of identity and query coverage) of the consensus sequence against the source genome. Unknown consensus sequences are mainly composed of short elements (median = 433 bp, Additional file 5: Fig. S2A) with medium–low copy numbers (median = 354 copies; Additional file 5: Fig. S2B). Though, it must be noted that for the well-analyzed species *Crassostrea gigas*, the percentage of unclassified elements drops down to 3.51% despite applying the same annotation pipeline (Additional file 4: Table S3). Overall, these results suggest that most of the unknown elements likely correspond to short, fragmented, or ancient families difficult to classify based on homology evidence alone.

The TE content also varied among and within different mollusc classes (Fig. 1A). The two TE-richest genomes were those of the pteriomorphian bivalve *M. philippinarum* (58.6%) and of the cephalopod *Octopus sinensis* (57.39%). Among bivalves, the mean TE content observed was 38.97%, with analyzed Pectinida showing a generally lower TE proportion with respect to all the other species (Fig. 1A). A significant positive correlation was observed between assembly size and TE content (Fig. 1B; Spearman’s rho = 0.72, *p* < 0.01).

### Class-level mollusc mobilome characterization using automatically generated TE sequence libraries

When analyzing the contribution of different TE classes in the overall transposon composition across all analyzed species (Fig. 1C), after excluding unknown elements, LTRs and SINEs resulted significantly under-represented compared to all other groups (Kruskal–Wallis rank test *p *< 0.05; pairwise Wilcoxon rank test with Bonferroni correction, *p* < 0.05), but no other significant differences were identified (Additional file 6: Fig. S3). The same pattern emerged when analyzing only bivalves, but they also showed a significant overrepresentation of DNA elements, including MITEs, over LINEs (Kruskal–Wallis rank test, *p* < 0.05; pairwise Wilcoxon rank test with Bonferroni correction, *p* < 0.05; Fig. 1D).

LINEs are ubiquitous elements and constitute the most common retroposon group (mean = 5.38%), but they were observed with a highly variable frequency, ranging from 1.15% in the polyplacophora *Acanthopleura granulata* to 24.78% in the gastropod *Achatina immaculata* genome, where they dominate the TE landscape. In bivalves, they represent from 1.30% of the host genome in the oyster *Crassostrea virginica* to 10.84% in *M. coruscus*. SINEs are present across all analyzed species but always in low copy number (mean = 1.69%) with a few, lineage-specific amplifications, such as in *Archivesica marissinica* (3.05%), Adepedonta order (*Sinonovacula constricta* and *S. grandis*, respectively 3.1% and 4.7%), the Arcida order (*Anadara kagoshimensis*, *Scapharca broughtonii*, and *Tegillarca granosa*; mean = 5.3%), in the Mytilidae *Bathymodiolus platifrons* (6.23%), and in the Polyplacophora *A. granulata* (4%). Also, LTR elements were generally found in low copy number in the analyzed species (mean = 1.52%), with the exception of the Unionidae species *Megalonaias nervosa* in which LTRs account for 6.66% of the host genome. We also observed a relatively high rolling circle (RC) element content (mean = 5.91%) associated with bivalve diversification, reaching an average of 12% among *Crassostrea* species and 9.69% in *Cyclina sinensis*. Notable exceptions to this trend are the two Unionida *Potamilius streckersoni* and *M. nervosa* in which RC elements are greatly reduced in the former (0.05%) and absent in the latter.

### General characterization of mollusc repeatome composition using automatically generated TE sequence libraries

**Fig. 2:**
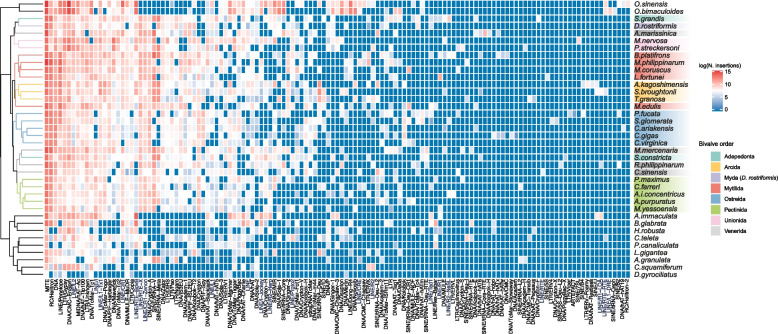
Hierarchical cluster analysis on the number of insertions for each transposon type. The insertion counts were obtained after defragmentation of the TE annotation with RepeatCraft on the RepeatMasker output obtained with the automatically generated TE sequence libraries (see the “Mining and annotation of interspersed repeats” section)

When clustering analyzed mollusc genomes based on the number of annotated insertions for each RepeatMasker transposon type (see the “Mining and annotation of interspersed repeats” section), we found that bivalves are clearly divergent from other molluscs, both when using a hierarchical (Fig. 2) and a *k*-mean clustering approach with 3 centers (Additional file 7: Fig. S4). However, when looking at the relationships between and within bivalve orders, a more complex scenario emerged, with lineages belonging to different orders intermingling with each other. The only exception to this pattern was the Ostreidae, whose clustering resulted in complete agreement with their known phylogenetic relationships [28].

Concerning LINEs, the elements L2, L1-Tx1, CR1, and I are the most ubiquitous types across molluscs with representatives in respectively 36, 35, 35, and 32 species, even though their genomic occurrence can vary to a great extent. The RTE-BovB type was found greatly expanded in cephalopods and in the gastropods *A. immaculata* and *Biomphalaria glabrata* compared to other species. On the contrary, RTE-X and CR1-Zenon elements were more represented in bivalve genomes but greatly reduced or even absent in cephalopods and gastropods*.* Finally, R2-Hero, R4-Dong, and CRE types are identified almost exclusively in cephalopods, with only R2-Hero found in low copy number in the *A. immaculata* genome and in some bivalve species but with a patchy distribution.

Multiple SINE lineages were found, belonging to V, Meta, Core, and MIR types, with V elements that can reach up to 4.3% in the Mytilidae *B. platifrons* genome*.*

Regarding LTRs, Bel/Pao, DIRS, and Ngaro types were mainly found in bivalves and in the gastropods *Lottia gigantea* and *Pomacea canaliculata*, although in low copy number, and they appeared almost absent in cephalopods. On the other hand, Gypsy and Copia elements are ubiquitous across all molluscs, with the former present in higher copy number.

For DNA elements, different types belonging to superfamilies Mutator-like elements (MULE), Mariner, PiggyBac, CMC, Mavericks, and hAT are present across all analyzed genomes. Kolobok, Zator, and Academ superfamily types are almost exclusively found in bivalves, while Zisrupton, Novosib, and Merlin superfamilies were found almost completely restricted to the analyzed cephalopods.

### Extraction and clustering of RT-containing LINEs

We decided to deeply and more confidently characterize the LINE complement by implementing an ORF-based extraction and classification approach (see the “ORF-based annotation of RT containing LINEs and Class II DDE/D elements” and “Tree-based classification of ORF-containing LINE elements” sections). Overall, we identified a total of 86,488 LINE loci exhibiting an RT domain in an ORF longer than 300 amino acids (Additional file 8: Table S4). These were grouped in 13,523 clusters following the 80–80 rule, and only 3601 of them were found composed of more than 5 elements, accounting for a total of 69,763 loci (80.7%). A great variation can be observed among species in terms of both diversity and richness of clusters. Among bivalves, *A. marissinica* genome resulted as the richest one in terms of RT-containing LINEs (6935 elements).

Overall, 8333 LINE loci (9.6%) were annotated as putative autonomous elements, here defined as insertions showing both RT and EN domains on the same ORF, longer than 300 amino acids and without interrupting stop codons (Additional file 8: Table S4). As expected, we found the number of LINEs with a RT domain being positively correlated with the number of identified putative autonomous elements (Spearman’s rho = 0.89, *p* < 0.01; Additional file 9: Fig. S5a). The assembly contiguity, here measured as the scaffold N50 value, was also found significantly correlated to the number of identified RT-containing LINEs (Spearman’s rho = 0.35, *p* < 0.05, Additional file 9: Fig. S5b) as well as to the number of identified putative autonomous elements (Spearman’s rho = 0.34, *p* < 0.05; Additional file 9: Fig. S5c).

### Phylogenetic analyses and classification of RT-containing LINEs

**Fig. 3:**
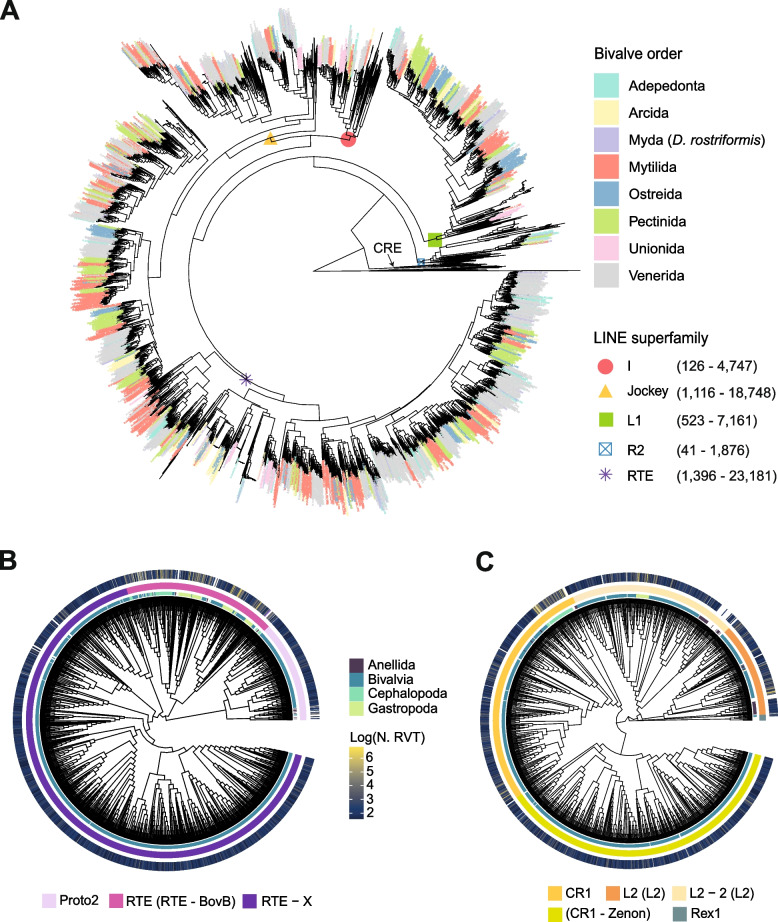
Phylogeny of mollusc LINEs. Phylogenetic analyses performed on extracted RT-containing LINEs (see the “Tree-based classification of ORF-containing LINE elements” section). **A** Maximum likelihood SupFAM tree #2 obtained by constraining the monophyly of different LINE superfamilies as recovered by the Neighbor-Joining topology (see the “Methods” and “Results” sections). Numbers in parentheses next to the LINE superfamilies represent the number of annotated clusters and the total number of elements represented by the included clusters, respectively. All tested trees with relative bootstrap values can be found in Additional file 29: Data S1. More detailed versions of the SupFAM tree #2 subtrees can be found in Additional files 13, 14, 15, 16, and 17: Figs. S7, S8, S9, S10, and S11. **B** RTE and **C** Jockey superfamily subtrees. The inner circle represents the taxonomic annotation of mollusc classes, and the mid one is the annotation of the different clades based on reference sequences extracted from RepBase and based on [29]. Note that the L2-2 clade includes Crack, Daphne, L2A, and L2B elements. Names in parenthesis refer to the RepeatMasker type classification. The outer circle shows the log scale number of elements grouped in each cluster. Reference sequences are represented by white spaces in the inner and outer circles

To classify the previously mined LINEs containing RTs in superfamilies and clades, we used a phylogenetic approach starting from amino acid consensus sequences built up from clusters with more than 4 members (see the “Tree-based classification of ORF-containing LINE elements” section). After the removal of poorly aligned sequences by TrimAl, 3252 LINE clusters were included in the phylogenetic analysis. We further added 259 reference sequences for classification purposes and annotated 111 other LINEs using RTClass1 (Additional file 10: Table S5). To obtain a reliable phylogeny of LINE elements useful for their annotation, we used both NJ and ML tree searches with and without topological constraints (Fig. 3A; Additional file 11: Fig. S6). When testing all topologies in a ML framework, we obtained the highest likelihood for one of the SupFAM tree (i.e., constraining the monophyly of all superfamilies as recovered in the NJ tree; SupFAM #2; Fig. 3A; Additional file 12: Table S6). Moreover, the obtained best tree also recovered more monophyletic clades compared to all other topologies (Additional files 13, 14, 15, 16, and 17: Figs. S7, S8, S9, S10, and S11) and resulted the most in agreement with both references [29, 30] and RepeatMasker/Dfam classification schemes. For these reasons, the SupFAM #2 tree was used for LINE classification as well as for all downstream analyses. Based on reference sequences, we managed to confidently classify all elements at the superfamily and clade level, except for 16 elements from *O. sinensis* genome, that were placed in a subclade of the I superfamily in a sister relationship with I-Loa-R1 and Tad1 reference sequences (unknown I clade; Additional file 16: Fig. S10). Moreover, as already shown (see [29]), L2A and L2B clades resulted to be paraphyletic, with polyphyletic Crack and Daphne elements clustering within them. For these reasons, henceforth, we will refer to these clades as L2-2 elements, while other elements will be simply indicated as L2. Interestingly, Proto2, RTE-X, and CR1-Zenon elements were only found in bivalves, with Proto2 also present in the annelida *Capitella teleta* (Fig. 3B, C). The complete phylogenetic tree-based annotation of all LINEs can be found in Additional file 27: Table S7. Generally speaking, the tree resulted in a complex branching pattern with multiple order-specific clades in each identified LINE clade/type, also highlighting multiple instances of expansion, contraction, and loss of LINE lineages in different bivalve orders (Fig. 3A; Additional filed 13, 14, 15, 16, 17: S7, S8, S9, S10, S11). Blastp against the full RepeatPeps library and RTClass classification widely confirm our phylogenetic tree-based annotation with only few discordances, which mainly concerned Proto2 elements classified as RTE-X.

### Richness, diversity, and distribution of RT-containing LINEs

**Fig. 4:**
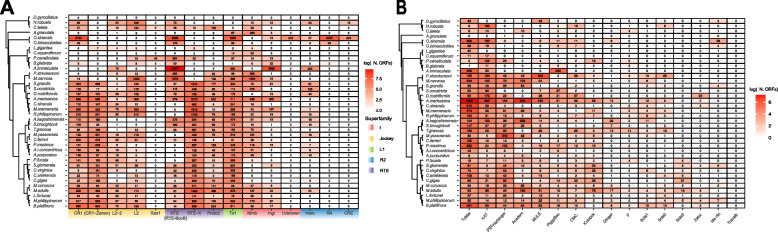
Richness of mollusc class II DDE/D-related transposons and LINEs. **A** Number of RT-containing LINEs annotated in each analyzed genome (see the “Tree-based classification of ORF-containing LINE elements”) and subdivided by clade following [29] or, when in parenthesis, by the RepeatMasker “type” classification. Clades are grouped by superfamily following Metcalfe and Casane (2014) and rresults from the SupFam tree #2 (Additional files 13, 14, 15, 16, and 17: S7, S8, S9, S10, and S11). “Unknown” refers to elements annotated based on an O. bimaculoides clade found nested in the I superfamily but missing any reference sequence (see Additional file 16: Fig. S10). Note that the L2-2 clade includes Crack, Daphne, L2A, and L2B elements. **B** Number of ORF-containing DDE/D-related transposons annotated in each analyzed genome and subdivided by superfamily following [31] (see the “ORF-based annotation of RT containing LINEs and Class II DDE/D elements” section)

We used Blastp against previously phylogenetic tree-based classified LINEs to annotate all clusters excluded from phylogenetic analyses (i.e., “low-copy number”, “singletons” and clusters removed by TrimAl; see the “ORF-based annotation of RT containing LINEs and Class II DDE/D elements” section; Fig. 4A; Additional file 18: Fig. S12). The RTE, Jockey, and L1 superfamilies were confirmed as the richest (*i.e.*, with more elements; Fig. 4A) and most diverse (*i.e.*, with more clusters; Additional file 18: Fig. S12) across molluscs. The only R2 elements found in bivalves were classified as Hero (363 elements). Nimb and Ingi clades are the only representatives of the I superfamily across molluscs, beside the Unknown clade coming from *O. sinensis*. The Rex1 clade was only found at a low copy number in the gastropods *P. canaliculata*, *B. glabrata* and the annelida *C. teleta*, while elements belonging to CR1-Zenon, RTE-X, and Proto2 lineages despite still being more highly represented in bivalves were also recovered at low copy numbers and/or with singleton elements in few non-bivalve species. Bivalve genomes exhibit a high diversity of LINE lineages, hosting members from 11 out of the 14 identified clades (CR1, CR1-Zenon, L2, L2-2, RTE-X, RTE-BovB, Proto2, Tx1, Nimb, Ingi, Hero). As a comparison, the gastropod *B. glabrata*, the cephalopod *O. sinensis*, and the ringworms *Helobdella robusta* and *C. teleta* showed eight different LINE clades. In *A. marissinica*, all clades, with the exception of Hero and L2-2, were expanded compared to other Venerida and Imparedentia. For Arcida, Pectinida, and Ostreida, we identified multiple instances of order-specific loss/contraction, such as the extreme reduction of the I superfamily in Ostreida (maximum of 9 members of the Ingi clade identified in *C. virginica*), of the RTE clade (RTE-BovB type) in Pectinida (11 members in *Chlamys farreri*) and the L2-2 clade in Arcida (only 1 element in *T. granosa*). The Unionida *M. nervosa* and *P. streckersoni* show notable differences in their LINE complement compared to all other bivalves, with a great reduction of the RTE-X and CR1/CR1-Zenon clades/type, which were found well-represented in other genomes, and an expansion of L2 and RTE-BovB elements in *M. nervosa*. The number of annotated RT-containing LINEs and the number of clusters were found significantly correlated for all superfamilies (Additional file 19: Fig. S13). Finally, the number of annotated autonomous elements is in line with previous results, but no member of the R2 superfamily was identified (Additional file 20: Fig. S14).

### Distribution of class II DDE/D-related transposons based on the number of identified ORFs

To classify ORFs derived from DDE/D-related transposons, we implemented an HMM-based approach starting from classified sequences from the 17 superfamilies described by [31] (see the “ORF-based annotation of RT containing LINEs and Class II DDE/D elements” section). Overall, we identify DDE/D class II-related transposons, with an ORF longer than 300 amino acids and no interrupting stop codons, coming from 16 out of the 17 superfamilies, for a total of 14,275 elements. Their distribution approximately recapitulates what we observed with automatically generated libraries (Fig. 4A). Specifically, the TcMar resulted in the richest superfamily in 21 species, accounting for 41% of the overall number of identified elements, followed by hAT, Academ, MULE, and PIF-Harbinger. Instead, Ginger, Sola1, Sola2, Sola3, Zator, Merlin, and Transib are less represented, with respectively 98, 95, 105, 64, 44, 63, and one element identified. Overall, bivalves possess at least one element across all superfamilies resulting in the most diverse mollusc group here analyzed in terms of the number of hosted DDE/D-related superfamilies, with Academ, Sola, and Zator elements that appear restricted to this clade. Interestingly, we found that *A. marissinca* genome hosts the highest number of DDE/D-related elements from the five superfamilies hAT, TcMar, PIF-Harbinger, Academ, and CMC compared to all other bivalves, similarly to what we observed for LINE elements.

### Construction of a manually curated library for LINE, SINEs, and DDE/D-related transposons

We used our annotated class I LINEs and SINEs and class II DDE/D-related ORFs in a “blast-extend-extract” approach to build a comprehensive and manually curated TE library of potentially or recently active elements for bivalves (see the “Manual curation of LINEs, SINEs, and DDE/D-related transposons” section). In total, we curated 840 LINEs, 119 SINEs, and 1018 DDE/D transposons for a total of 1917 elements. These libraries were reduced respectively to 810, 37, and 762 families after CD-HIT clustering.

For the LINE library, all consensus sequences possess a RT domain, while we manage to reconstruct a RT + EN segment for 740 (91%) of them. Therefore, although we did not systematically search for full-length elements due to frequent 5′ truncations, most of these families may correspond to potentially active or recently active elements for which exist copies across the genome with recognizable RT and EN domains. It must also be noted that only clusters that exhibit at least one copy with an RT and EN domain on an ORF longer than 300 amino acids were selected for manual curation (see the “Manual curation of LINEs, SINEs, and DDE/D-related transposons” section). The length of the resulting consensus sequences ranges from 1786 to 9087 bp with a mean of 5023 bp. As expected, different LINE superfamilies show different length distributions (Additional file 21: Fig. S15) with members of I and L1 superfamilies being generally longer (mean = 6122 bp and 5851 bp, respectively), followed by Proto2 (mean = 5675 bp), CR1-Zenon (mean = 5204 bp), RTE-X (mean = 4937 bp), L2 (mean = 3991 bp), CR1 (mean = 3791 bp), and RTE-BovB (mean = 3583 bp). These values largely recapitulate the canonical length of full-length elements described in the literature, as for RTE-BovB (3.2 kbp) and L1 (6–8 kbp) [32, 33], proving a successful implementation of the “blast-extend-extract” approach.

The length of SINE elements varies between 174 and 404 bp (mean = 307 bp). Nine of them were classified as V elements, eight as Meta, eight as MIR, four as Deu, and one as Core, while the other seven elements lacked a family-level classification.

For DDE/D-related elements, after checking for TIRs, flanking TSDs, and the presence of an ORF longer than 300 amino acids with a significant hit against DDE/D-related HMM profiles, all curated consensus sequences correspond to autonomous full-length consensus elements. Specifically, our library includes: 332 TcMar, 133 hAT, 100 Academ, 58 PIF-Harbinger, 43 Kolobok, 39 MULE, 27 PiggyBack, 14 Sola2, eight Sola1, three Zator, three Merlin, and two CMC transposons. Also in this case, their length greatly varies between different superfamilies (Additional file 22: Fig. S16) and results concordant with known estimations [34] with Sola1 (mean = 5445 bp) and Academ (mean = 5565 bp) being generally the longest elements, and Merlin (mean = 1635 bp) and TcMar (mean = 1935 bp) being generally the shortest one.

### Evolutionary and expression analyses of curated LINE and SINE families

**Fig. 5:**
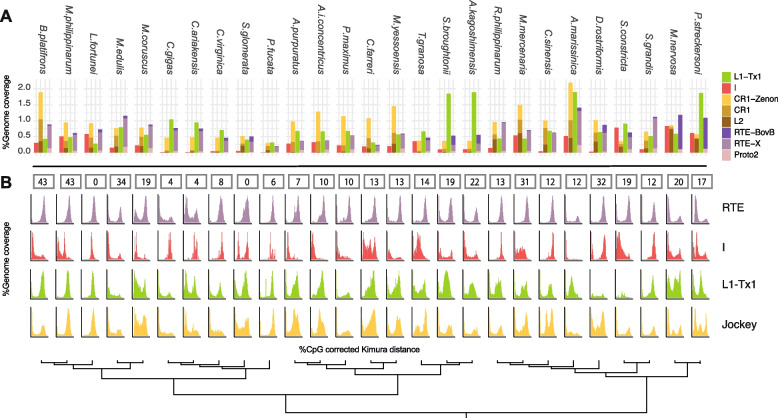
Genome occurrence and evolutionary history of manually curated LINE families. RepeatMasker results obtained using our manually curated set of LINEs (see the “Manual curation of LINEs, SINEs, and DDE/D-related transposons” and “Genome annotation of LINEs and SINEs using manually curated libraries and phylogenetic inference of curated LINE families” sections). **A** Genome coverage of curated families for each LINE clade/type. **B** CpG-corrected Kimura distance of each insertion from its consensus sequence as a proxy for the time of the transposition event for each LINE superfamily. The *X*-axes range from 0 to 50 while the *Y*-axes are on different scales for each specie/superfamily and represent the relative genome coverage. Numbers above the graphs represent the number of families for each species that possess insertions both in recent time (divergence < 5) and in the past (divergence > 30) requiring at least 30 annotated insertions in the recent divergence bin and 5 in the old one. Only 3′-anchored insertions longer than 100 bp were considered for this latest purpose (see the “Genome annotation of LINEs and SINEs using manually curated libraries and phylogenetic inference of curated LINE families” section)

We used the previously curated LINE library to analyze the evolutionary dynamics of potentially active or recently active LINE families (see the “Transcription potential of curated LINE families” section). First, the number of curated families and the number of putative autonomous elements were positively correlated to each other (Spearman’s rho = 0.88, *p* < 0.01), suggesting their representativeness of the overall LINE complement. Phylogenetic analyses of curated families reflect what we observed in the full LINE tree, with elements found in the same host genome characterized by long branches and intermingling with those found in other species, even belonging to different bivalve orders (Additional files 23, 24, 25, and 26: Figs. S17, S18, S19, and S20). After masking the genome with RepeatMasker and both LINE and SINE curated libraries, the genomic occurrence of curated families ranges from < 2% in the pectinida *C. farreri*, in the oysters *Pinctada fucata*, *Saccostrea glomerata*, and *C. virginica,* and in the arcid *T. granosa,* to > 4% in the Unionida species *A. marissinica* and *P. streckersoni* (Fig. 5A)*.* It must be noted that these estimations are only based on potentially active or recently active families that were selected for manual curation and therefore should not be considered as an estimate of the overall LINE complement.

Repeat landscape showed similar activity profiles for CR1/Jockey, L1, and RTE superfamilies across the majority of analyzed bivalves, with one or two bursts of activity localized at low (1–5%) but also at high (30–50%) divergence from the consensus. However, coherently with the distribution of LINE clades, some bivalves lack the recent peak of activity (Fig. 5B). In other instances, recent lineage-specific expansion of different LINE clades/types can be observed, such as for RTE-BovB in Unionida and CR1, CR1-Zenon, and RTE-X in *A. marissinica*. Moreover, using high-confidence 3′-anchored insertions (i.e., insertions aligning within the first 50 bp of the 3′ end of the consensus and longer than 100 bp), we found a variable number of ancient LINE families that showed both recent (at least 30 copies with less than 5% divergence) and ancient (at least 5 copies with more than 30% divergence) activities (Fig. 5B). All oysters as well as *L. fortunei*, *P. fucata*, and *Argopecten purpuratus* possess between zero (*L. fortunei*, *S. glomerata*) and eight (*P. fucata*) ancient families while in all other bivalves, their number can range between 10 in *Pecten maximus* and 43 in the Mytilida *B. platifrons* and *M. philippinarum*. We assess the transposition potential of curated families by mapping gonad-derived RNAseq reads on 3′-anchored insertions longer than 3 kbp and extracted from five bivalve genomes. Specifically, we found 96, 383, 1054, 346, and 801 insertions useful to map RNAseq reads in respectively *C. farreri*, *C. gigas*, *Mercenaria mercenaria*, *Mizuhopecten yessoensis*, and *S. constricta*. The obtained transcription levels (estimated as transcripts per million (TPM)) per family were then tested for a correlation with the number of 3′-anchored insertions longer than 100 bp (to allow the presence of 5′ truncated copies) of the corresponding family. Across all analyzed species, tissues, and biological replicates, we found a significant, positive correlation between the number of insertions and the per-family transcription level (Spearman’s rho = 0.48 to 0.70, all *p* < 0.01; Table 1), a pattern consistent with an ongoing transposition of these elements [35].

**Table 1:** Spearman’s rho correlation coefficients between family-based LINE transcript levels and number of insertions

Species	FG_1	FG_2	FG_3	MG_1	MG_2	MG_3
*C. farreri*	0.54	0.60	0.60	0.50	0.48	0.52
*C. gigas*	0.46	0.50	0.50	0.40	0.48	0.51
*M. mercenaria*	0.64	0.61	0.60	0.57	0.60	0.60
*M. yessoensis*	0.70	0.68	0.67	0.63	0.60	0.62
*S. constricta*	0.56	0.53	0.59	0.66	0.61	0.56

Transcript levels were calculated as log2-transformed transcripts per million (TPM). Only insertions longer than 3 kb were used for mapping RNAseq reads (see the “Transcription potential of curated LINE families” section). Each tissue and biological replicate was separately tested for each species

All *p*< 0.01

*MG* male gonads, *FG* female gonads

We also added SINE families in the same RepeatMasker run, and we obtained their reliable genome occurrence in the 13 species selected for in-depth SINE mining (see the “Richness, diversity, and distribution of RT-containing LINEs” and “Construction of a manually curated library for LINE, SINEs, and DDE/D-related transposons” sections). SINE genome occurrence can greatly vary between and within species belonging to different bivalve orders (Table 2). The genomes of *A. marissinica* (6.02%), *T. granosa* (3.69%), *S. broughtonii* (4.37%), and *B. platifrons* (4.68%) host a relatively high number of SINEs while on the contrary, we observed a great reduction in the genome of *C. gigas* (0.08%) and *S. glomerata* (0.31%). Different SINE types successfully colonize different bivalve genomes: the Deu family was found to be dominant in *A. marissinca* (72% of the overall SINE complement), *C. sinensis* (94%), and *S. broughtonii* (55%), while the V family is dominant in the *B. platifrons* genome (67%) and the Meta in *S. constricta* (54%) and *S. grandis* (50%). Finally, in *T. granosa*, both Deu and V families occupy a considerable proportion of the overall SINE complement of respectively 30% and 46%. Finally, we did not find any evidence of a significant correlation between SINE and LINE genomic occurrence (Spearman’s rho = 0.31, *p* = 0.33).

**Table 2:** Percentage of genome occurrence of different SINE types in the 13 selected bivalves

Species	Meta	Core	Deu	V	Unknown	MIR	**TOT**
*A. marissinica*	0.22	> 0.01	4.1	1.4	0.3	> 0.01	**6.02**
*C. sinensis*	0.03	0.04	2.7	0.01	0.08	/	**2.86**
*C. gigas*	> 0.01	/	/	0.04	/	0.04	**0.08**
*S. glomerata*	0.3	/	> 0.01	> 0.01	/	> 0.01	**0.31**
*T. granosa*	0.8	> 0.01	1.13	1.7	0.05	> 0.01	**3.69**
*S. broughtonii*	0.1	> 0.01	2.42	1.4	0.41	0.04	**4.37**
*M. coruscus*	0.16	0.01	0.5	0.3	> 0.01	> 0.01	**0.97**
*B. platifrons*	1.48	> 0.01	> 0.01	3.19	/	/	**4.68**
*S. constricta*	1.34	> 0.01	> 0.01	1.08	0.01	0.04	**2.47**
*S. grandis*	1.34	> 0.01	> 0.01	0.94	0.35	0.04	**2.67**
*M. yessoensis*	0.2	/	> 0.01	0.35	> 0.01	0.26	**0.81**
*P. maximus*	0.23	/	> 0.01	0.50	/	0.04	**0.77**
*M. nervosa*	0.99	/	> 0.01	0.75	0.11	0.11	**1.96**

## Discussion

### A comprehensive TE annotation for bivalves

The phylum Mollusca shows a high level of organism diversity and includes species that are important for both their ecological and economic value. Although genomic studies are accumulating and comparative analyses are becoming more common for these organisms, a deep analysis of the mobilome is still limited to single genomes or to a few comparative studies with only a handful of species [24, 25]. As could be expected, this also resulted in a scarce representativity of molluscan TEs in the public databases which makes their automated annotation less reliable. As previously shown, high-quality, manually curated repeat libraries are considered necessary for a consistent, reliable repeat annotation and characterization in novel genomes [26, 27]. In the present analysis, we decided to focus our efforts on bivalves, which represent 27 out of the 39 analyzed genomes, due to the recent, increasing genome sequencing efforts for this class. The inclusion of five gastropod genomes, representative of their major lineages, together with two cephalopods, one polyplacophoran genome, and three annelids allowed us to identify the major shifts in TE composition that occurred during molluscan evolutionary history. To overcome the limitations of automatically generated TE sequence libraries, we set up a pipeline which included both automated, ORF-based extraction and classification and manual curation approaches and that has been used consistently across the analyzed genomes. In particular, the manual curation process allowed us to provide the first freely available and manually curated repeat library for bivalves, comprising DDE/D, LINEs, and a subset of SINE elements for a total of 1609 elements comprising all identified LINEs, with the exception of the low copy number R2 superfamily and 12 different DDE/D-related superfamilies. These new genomic resources could help future genome annotation projects and shed novel insight into TE evolutionary dynamics in bivalves. On the other hand, the ORF-based approach allows us to confidentially characterize both LINEs and DDE/D-related TE complements. As a comparison, concerning LINEs in the RepBase library v. 20181026, 1031 sequences are deposited for molluscs, with 796 of them belonging to well-characterized *C. gigas*. Fifty-nine of these are annotated as LINEs and, more specifically: one R2, two CR1, 12 CR1-Zenon, 14 L1-Tx1, and 27 RTE-X. In the present analysis, we also found multiple Proto2, RTE-BovB, and L2/L2-2 elements. Regarding DDE/D transposons, out of 422 total sequences coming from RepBase for *C. gigas*, 92 possess an ORF longer than 300 amino acids, and they belong to 13 different superfamilies. With our approach, we manage to identify ORF-derived signatures coming from all of them, with the expectation of Zator, Merlin, and Sola1 for which only two sequences are deposited for each superfamily in RepBase. Overall, these results suggest that our ORF-based approach successfully captures in a flexible way most of the diversity of coding TEs in non-model species.

We also paid particular attention to filter out possible misannotations from the automatically generated TE sequence libraries, such as the inclusion of repetitive genes, tandem repeats, degenerate, and low-copy number families, which are hard to correctly annotate and classify. This approach is probably quite conservative, indeed in some instances, it provided different estimates of the overall TE content compared to published genome papers. For example, in *Mytilus edulis*, our study estimated 47% of TE content vs the 56% provided in [36]; the same holds for *S. glomerata* (42% in the present study vs 45% in [37]) and for *A. granulata* (18% here vs 23% in [38]). In other instances, though, our analysis provided almost the same estimates as in the previous analyses, as in *M. coruscus* (49% here vs 47% in [39]), *A. immaculata* (41% here vs 40% in [40]), and *M. mercenaria* (51% here vs 49% in [41]).

TEs have been shown to be one of the major contributors to genome size evolution in metazoan lineages, such as insects [42] and vertebrates [43], and in angiosperms as well [44]. Our analyses provided further support for this hypothesis finding a positive correlation between TE content and assembly size also in molluscs. Across bivalves, the TE content varies greatly, ranging from ~ 20% in the Pectinida *M. yessoensis* up to ~ 60% in the Mytilida *M. philippinarum*. Different sequencing technologies and sequencing depths could potentially contribute to such differences; however, it must be noted that also for Illumina sequenced genome, we observed a high TE content, such as for the M. *philippinarum* and *B. platifrons*. It is interesting that the low TE load found across all analyzed Pectinida species. In fact, this order includes the most TE-poor bivalve species, with almost twofold less TE content compared to Mytilida and Ostreida. Similar occurrences of interspersed repeats were already observed for this lineage during whole genome sequencing projects [45–48], and transposable elements hosted by *M. yessoensis* were found to be generally less active in recent times compared to what was observed in the Pacific and pearl oysters [46]. This low TE activity was suggested to be the reason behind their conserved genome architecture that could resemble that of bilaterian ancestors [46]. However, as well described in birds, low TE content and apparent lack of activity could also originate from nonallelic homologous recombination which could physically remove TEs and other repetitive regions from the genome without implying a general genomic stability [49].

Concerning class I elements, LTR elements in general occupy a low proportion of host genomes as previously observed by [24], while we found LINE elements as the richest retroelements. They contribute from 1.61 to 10.84% respectively in *C. virginica* and *M. coruscus* genomes using automatically generated TE sequence libraries and between 6.18% for *A. marissinica* and 0.82% for *P. fucata* using manually curated libraries. A similar scenario occurs also for SINE elements, whose genome coverage can greatly vary between different bivalve species using both automatic and manually curated libraries. In both instances, we identify the genomes of *A. marissinica*, *B. platifrons*, and Arcida as richer in SINEs compared to other analyzed bivalves, but we did not find any evidence of a general increase of the SINE complement coupled with an increase of their autonomous counterparts LINEs.

Class II and RC elements generally outnumber other TEs, especially in bivalves where DNA elements were found significantly enriched compared to all retroposons. This is strikingly different from what is observed in mammals, where retroposons constitute the most successful TE group, but similar to what is observed in actinopterygian fishes where class II elements greatly dominate the overall TE content [43]. Moreover, we found that non-autonomous counterparts (MITEs) occupy a considerable proportion of host genomes suggesting the high proliferation of small, non-autonomous copies. Within the most rich superfamilies of DDE/D ORF-derived signatures in bivalve genomes, we identified TcMariner and hAT lineages. Interestingly, the same superfamilies were also found to be the richest of ORF signatures in all other analyzed molluscs and to be ubiquitous even when using the automatically generated TE sequence libraries. Both TcMar and hAT superfamilies were found anciently expanded across cephalopods in a recent study from [25], possibly suggesting their high representativeness as a plesiomorphic state of molluscs. On the other hand, we could identify notable examples of bivalve-specific expansion, such as for Academ and RC elements. The former seems to be poorly represented in non-bivalve genomes, with only few ORF identified in the ringworms *C. teleta* and *H. robusta* and few insertions annotated in non-bivalve molluscs when using automatically generated libraries. RC elements can occupy up to 12% of the analyzed *Crassostrea* species. As a comparison, RC have a more patchy distribution in arthropod genomes, generally contributing to a smaller extent of the genome size with only few lineage-restricted expansions (e.g., *Drosophila* and *Musca domestica* [42, 50]). Also in plants, where they were first discovered, they are usually less represented, covering a maximum of 6% of the maize genome [51].

### A highly diverse TE repertory characterizes bivalve molluscs

Both hierarchical and *k*-means clustering using automatically generated libraries clearly separated bivalves from other molluscs highlighting important differences in their TE complement. Although the scenario among these taxa appeared more complex, with the only case of full intra-order agreement between clustering analyses and species phylogeny in Ostreida, the analyses of both LINEs and DDE/D elements provided some notable examples of lineage-specific element differentiation.

Similarly to what has been observed in *Drosophila* [52], fishes [43, 53, 54], and other non-mammalian vertebrates [33, 55], we found that bivalves are characterized by a highly diversified DDE/D and LINE complement. For the former, we identified ORF-related signatures coming from 17 different superfamilies while for LINEs, we found 11 clades coming from all known superfamilies. Notable cases are the emergence of RTE-X, Proto2, and CR1-Zenon elements which, similarly to DDE/D Academ, appear almost limited to bivalve molluscs. Moreover, the presence of multiple, order-specific clusters across the LINE phylogeny, especially within Jockey and RTE superfamilies, suggest that these elements were already greatly diversified before the fast radiation of bivalves that occur in the early Ordovician, around 499 Mya [56, 57]. It is worth noting the underrepresentation of R2 elements across all molluscs (with exception of *O. sinensis*), a pattern strikingly different from what has been observed in other major lineages like arthropods, which are among the most successful LINEs [8, 42]. The Hero clade seems to be the only R2 element present in bivalve ancestors and the only identifiable in extant species, even though we could not identify any autonomous element.

Horizontal transposon transfer (HTT) can be a major source for the emergence of lineage-specific TE repertories, especially for aquatic species [23, 58]. In bivalves, the most studied transposon, the LTR element Steamer—a retroposon initially linked to transmissible fatal leukemia-like disease [59]—is involved in multiple HTT events [23]. The contribution of HTT in the evolution of TE repertories can be exceptionally important for DNA transposons, while LINE elements are thought to be generally transmitted through vertical inheritance [58, 60, 61]. Indeed, contrary to DNA transposons, proteins encoded by retroposons highly favor the transposition of the RNAs from which they are encoded [62]: this *cis* preference is thought to allow their long-term persistence under vertical transmission, even with the simultaneous presence of multiple, non-autonomous copies [62]. However, multiple cases of HTT involving LINE elements have been described in the literature, also involving bivalves and other aquatic species [63]. Moreover, HTT events involving Harbinger elements between bivalves and sea kraits have also been recently described by [64]. Here, we have not interrogated our dataset for such events, and therefore, their impact on the overall evolution of bivalve TE complement, and especially of DNA elements, remains poorly explored. However, our curated set of LINEs and full-length DDE/D transposons, together with the continuous rapid increase of novel genomic resources for bivalves, could represent an additional important starting point for future works.

### Different bivalve orders are characterized by different LINE clades

The highly diverse ancestral bivalve LINE complement appeared to undergo multiple lineage-specific rounds of amplification and extinction/reduction events coupled with the diversification of major bivalve orders. Worthy of attention are the cases of the Unionida *M. nervosa* and *P. streckersoni* and of the chemoautotrophic symbiont-hosting *A. marissinica*. *M. nervosa* and *P. streckersoni* are characterized by an increased genome coverage of RTE-BovB elements (RTE clade) compared to other bivalves. Moreover in *M. nervosa*, we identified 121 RTE-BovB autonomous elements, accounting for 44% of the total number of autonomous RTE-BovB identified across all analyzed bivalves. At the same time for both species, we observed an apparent contraction of the bivalve-rich RTE-X, CR1, CR1-Zenon, and RC complements, a pattern found uniquely in this order. We can speculate that this drastic change in TE repertories could be due to their ancestral colonization of freshwater environments. Indeed, Unionida are an ancient, whole order of freshwater-only bivalves [65], and they are characterized by unique life history traits such as parental care and larval parasitism [66]. The colonization of new ecological niches and/or possible related founder effects could drive drastic changes in TE content both due to alteration in the efficiency of natural selection and due to the impact of genetic drift with the stochastic loss and survival of different TE lineages [67, 68]. Similar cases of rapid LINE expansion due to genetic drift have also been observed in birds [69]. A similar scenario could also potentially occur for the deep-sea chemoautotrophic, symbiont-hosting *A. marissinica*. In this species, all LINE clades appeared expanded, with a peak of activity near the present for all superfamilies and likely driven by the high amount of hosted autonomous elements (*N* = 799) coming from 9 out of the 11 clades. Moreover, it also hosts a high number of DDE/D-related transposons compared to other bivalves. Our findings are coherent with a suggested increased TE activity coupled with the diversification of pliocardiines (~ 70 Mya [70]). On the other hand, we could observe multiple cases of loss/reduction of LINE representatives, for example in Ostreida (I superfamily), Pectinida (RTE-BovB clade), and Arcida (L2 and L2-2 clades).

Interestingly, oysters seem also to be generally depleted of SINEs, while Arcida, *A. marissinica*, and *B. platifrons* appeared enriched. As suggested by the absence of correlation between overall LINE and SINE genome coverage, a general estimation of the representativity of hosted LINEs is not sufficient to explain the overall variation in the genome occurrence of their non-autonomous counterparts. Until now, for only three out of eight different SINE families described in bivalves so far, their LINE donors [15] have been identified, and our curated LINE library could represent an important starting point for future analyses aimed to elucidate their co-evolutionary dynamics.

### Contemporary activity and long-term survival of multiple and diversified LINE lineages in bivalves

Analyzing autonomous elements, we identified multiple and diversified LINE lineages belonging to different clades that, although accounting for a relatively small proportion of the genome, co-exist within the same host. Moreover, the analysis of manually curated families showed that they may effectively be able to replicate and jump, as highlighted by the recent peak of activity identified in the repeat landscape analyses, by the presence of multiple elements showing both RT + EN domains, and by the significantly positive correlation that we found between family-level transcription levels and a number of insertions. Indeed, we expected to find a significantly higher amount of TE copies for highly transcribed families only when one or multiple elements are effectively able to overcome host mechanisms of post-transcriptional silencing (e.g., RNA interference). These patterns are strikingly different from what is observed in mammals where only one or few families are active at a given time and a handful of L1 lineages account for almost 20% of their genome but, again, matching what is observed in fishes and other non-mammalian vertebrates where LINEs are less dominant [55]. This mammal-specific evolutionary model is often referred to as an arm race between the host and the elements, and one of its landmarks is a cascade structure of the LINE phylogeny, where highly active elements are fastly replaced by new ones [33, 71, 72]. On the contrary, our results, together with the general lack of species-specific clusters with short branch lengths and a high number of copies in the LINE phylogenetic trees, could highlight a reduced mobilization with multiple, less harmful “stealth drivers” that occasionally emerge as for the previously discussed RTE-BovB elements in Unionida [33, 72–75]. At the same time, this pattern could also be explained by the high turnover of LINE copies due for example to ectopic recombination [33, 39] and/or lower fixation rate of recently mobilized elements [55]. Different TE evolutionary models are thought to be responsible for the different repression mechanisms adopted by the host to control transposition activity [55]. Indeed, in the arm race scenario, the host organism must quickly counteract highly active TEs through the evolution of sequence-specific repressors to limit their deleterious effect. On the contrary, a more TE-diversified genome with multiple stealth drivers’ elements could be more efficient in a general process, like methylation, rather than a sequence-specific mechanism. Coherently, bivalves are characterized by a high diversity not only of LINEs but also of class II elements and by high levels of methylation [76, 77] which could, therefore, represent the main repression mechanism.

Interestingly, across RTE, Jockey, and L1, we identified an additional ancient burst of activity that seems to be shared between multiple species, and multiple families were found to be active both in recent times and in the past. The ancient origin of bivalve orders makes it difficult to claim a shared activity without knowing their substitution rates, and even in that case, substitution saturation can obscure ancient activities. Nevertheless, the presence of both recent and ancient peaks underlies the long-term survival of these LINE lineages, as also visible in the phylogenetic trees of curated autonomous families, where their emergence tends to precede the speciation event of the host. Overall, these findings are coherent with the stealth driver model that allow TE lineages to “silently” survive over evolutionary timescales and occasionally emerge due to weakened genomic defenses, as reported in a narrower scale for the *Drosophila nasuta* species group [78], suggesting a possible important role of these elements in shaping both recent and more ancient phases of bivalve diversification.

## Conclusions

In the present study, we performed the first comparative analysis of transposable element evolutionary dynamics across molluscs with a particular emphasis on bivalves, an ecologically and economically important group. Despite genomic resources still being limited to few representative species compared to other clades, such as insects, the relatively low taxon sampling allowed us to deeply characterize for the first time their LINE and class II DDE/D-related complement. Moreover, because a high-quality repeat library is essential for the analyses of new genomes, our reference set of classified LINEs and DDE/D elements can be used to improve genome annotations and/or to easily classify novel elements across other lophotrochozoans. We also want to emphasize the necessity to extend similar analyses to other classes of transposons, empowering the scientific community with novel and high-quality genomic resources. While TEs have been hypothesized to be involved in the evolution of multiple bivalve genomic oddities, such as high levels of gene presence-absence variation [79] and of hemizygosity [80], the ability to identify their possible role deeply and consistently in shaping bivalve genome evolution will be limited as long as the great majority of elements are unclassified, fragmented, or not freely accessible for the scientific community.

With our approach, we discovered a diverse set of LINEs and DDE/D that were likely already greatly diversified in the most recent common ancestor of bivalves. The restricted emergence of the bivalve-rich Proto2, RTE-X, CR1-Zenon, and Academ elements could have contributed to bivalve fast radiation providing novel raw genomic material for their diversification. Moreover, we found that this LINE diversity seems to be maintained across extant species by an equally diverse set of potentially contemporary active families that could follow a stealth driver model of evolution. Indeed, multiple families seem to be able to survive and co-exist for a long period of time in the host genome without triggering the evolution of sequence-specific repression mechanisms, resembling what was previously observed in multiple non-mammalian vertebrates such as lizards and fishes. Finally, despite their relatively low genome occurrence, several LINE superfamilies/clades/types emerged, and others contracted in a lineage-specific manner during the diversification of bivalves. Therefore, this highly diverse LINE complement, despite being less represented than class II elements, is a rather dynamic portion of bivalve genomes and can play important roles in local adaptations and lineage-specific evolutionary dynamics.

## Methods

### Genomic resources and phylogeny construction

Thirty-six molluscs and three annelid genomes were downloaded from publicly available resources (NCBI, GigaDB, Dryad, MolluscDB, dbSROG, and Phaidra, see Additional file 1: Table S1), giving preference to bivalve assemblies representative of their major clades. Concerning molluscs, we selected 27 genomes belonging to bivalves, five to gastropods, two to cephalopods, and one to the polyplacophoran *A. granulata*. The species tree was manually reconstructed following the phylogenetic relationships found in recent phylogenomic studies [81–84] as well as the reference phylogeny presented in MolluscDB [85].

### Mining and annotation of interspersed repeats

For each analyzed genome, we compiled species-specific repeat libraries using a combination of structural and homology-based methods. RepeatModeler v. 2.0.1 [86] with the LTR pipeline extension which includes the structural-based LTRharvest [87] and LTR_retrivier packages [88], MITE Tracker [89], and HelitronScanner v. 1.1 [51] were used to build de novo consensus libraries. All software were run with default options except for HelitronScanner for which we increased the threshold of the minimum match score for both 5′ and 3′ ends from the default 5 to 10: this increases the specificity (*-ht* and *-tt*) despite decreasing the sensitivity. RepeatModeler consensus sequences were classified based on RepBase (v. 20181026) and Dfam (v. 3.1) databases, whereas MITEs were not further classified and considered only as non-autonomous DNA elements.

Bivalve genomes are characterized by high levels of duplicated genes, especially across immuno-related families (e.g., [84]) as well as by segmental duplications [90–92]. To reduce the possible inclusion of non-TE-related consensus sequences, the species-specific libraries were cleaned to remove (a) non-TE-related genes and gene fragments, (b) tandem repeats, (c) redundancy, and (d) low copy number repeats. For the first purpose, we started cleaning the reference proteomes of *H. robusta* (GCF_000326865.1), *P. canaliculata* (GCF_003073045.1), *L. gigantea* (GCF_000327385.1), *O. sinensis* (GCF_006345805.1), *M. yessoensis* (GCF_002113885.1), *C. gigas* (GCF_902806645.1), *C. virginica* (GCF_002022765.1), and *P. maximus* (GCF_902652985.1) from possible TE-related proteins. Blastp (*E*-value < 1E − 10) was used against a reference set of transposon-related proteins covering all TE classes and obtained from the EDTA package [93] and the Repeatpeps library from the RepeatMasker package [94]. Putative TE proteins were removed, and the resulting protein set was used as a database for blastx (*E*-value < 1E − 10) searches of our repeat libraries. Finally, ProtExcluder v. 1.1 [95] was used to remove non-TE-related genes and gene fragments. For purpose (b), we used the *cleanup_tandem.pl* script from the EDTA package requiring a minimum length of the consensus sequence after removing tandem repeats of 50 bp and a minimum percentage of non-ambiguous characters greater than half of the consensus length. Cleaned libraries were merged with 1031 consensus sequences from the Mollusca RepBase library, and (c) redundancy was reduced using CD-HIT [96] following the 80–80 rule (i.e., requiring a minimum 80% identity along the 80% of the shortest sequence [7]) with the parameters: *-c 0.8 -n 5 -aS 0.8 -g 1 -G 0 -t 1*. For the last step (d), each species-specific non-redundant library was searched with blastn against the corresponding genome with a required minimum query coverage and identity of 0.7. Sequences with less than 5 hits were removed to construct our final set of consensus sequences (i.e., 38 species-specific repeat libraries).

Annotation of repeats in each analyzed genome was achieved with running RepeatMasker v. 4.1.0 in sensitive mode (*-s*) using each of the species-specific repeat libraries as a custom database for the corresponding genome, without searching for low complexity repeats (*-nolow*) and small RNA (*-norna*). To improve the repeat annotations, the RepeatMasker output files were post-processed with RepeatCraft [97] in *loose* mode to merge closely related genomic fragments belonging to non-overlapping regions of the same consensus sequence. A hierarchical and *k*-means clustering of the number of TE insertions was performed respectively with the ComplexHeatmap R package v. 3.12 (Kendall’s *τ* clustering method) and the *kmeans* function specifying 3 centers. A flowchart describing the whole workflow is presented in Additional file 2: Fig. S1.

### ORF-based annotation of RT containing LINEs and class II DDE/D elements

To have a more precise picture of the representation of different superfamilies and clades of both LINEs and DDE/D class II elements, we applied an ORF-based extraction and classification pipeline. Firstly, insertion sites resulting from RepeatCraft analyses were extracted with the *bedtools* suite [98] together with 1000 bp at both ends to correct for possible partial/fragmented annotations due to the likely incomplete status of automated generated consensus sequences [26]. ORFinder was then used to identify and extract non-overlapping open reading frames (*-n*) with a required methionine as the start codon and a minimum ORF length of at least 300 amino acids (i.e., 900 nucleotides; *-ml 900*). To further characterize both class II DDE/D-related transposons and LINE elements, we used an HMM-based approach. For the former, we started from the amino acid sequences corresponding to DDE/D domains found in the 17 superfamilies described in [31]. All sequences coming from each superfamily (namely hAT, Tc1/Mariner, PIF/Harbinger, CMC, Merlin, MULE, P, Kolobok, Novosib, Sola1, Sola2, Sola3, PiggyBac, Transib, Academ, Ginger, Zator) were downloaded and separately aligned with MAFFT v. 7.475 [99] (*E-INS-i* strategy), and from each alignment, we build up a superfamily-specific HMM profile using the *hmmbuild* function from the HMMER3 package [100]. The collection of all 17 profiles was then used as a target database for *hmmscan* homology searches (*E*-value < 1E − 5) against all extracted ORFs provisionally annotating each element based on the corresponding best hit. To avoid misclassification of Ginger elements due to their high homology to *Gypsy*-encoded integrases [101] and to confirm the classification of all ORFs, we additionally blasted all significant hits against the full RepeatPep library (Blastp; *E*-value 1E − 05), imitating a reciprocal best-hit approach. Sequences with a best hit against a different superfamily compared to our previous HMM-based classification were considered as miss-classified and discarded.

For LINE elements, we started with an RPSblast search on the same set of extracted and translated ORFs against the complete CDD database (*E*-value < 1E − 05). Sequences with a significant hit against RT-related profiles were considered as putative retrotransposons (see Additional file 28: Table S8 for a list of CDD entries). To distinguish between LTR- and LINE-derived RT-containing ORFs, all LINE and LTR elements from the Repeatpeps library were extracted and separately aligned with MAFFT v. 7.475 (*l-INS-i* strategy) together with the seed sequences of the RVT_1 Pfam HMM profile (PF00078) to manually identify boundaries of the RT domain. We extracted LINE and LTR RTs from the resulting alignments, and we built two class-specific HMM profiles with the *hmmbuild* function from the HMMER3 package. The two profiles were then used as a target database for *hmmscan* (*E*-value < 1E − 5) homology searches of our previously identified RT-containing ORFs. Sequences with the best hit against the LTR-specific RT profile were considered as putative LTR and therefore discarded from subsequent analyses. LINE elements were considered autonomous when both RT and EN domains (see Additional file 28: Table S8 for a list of CDD entries) were present on the same ORF (i.e., non-intervening stop codons). Sequences missing the EN domain were classified as RT-only LINEs.

To test the interplay between assembly quality and the ability to identify RT-containing and autonomous LINEs as well as DDE/D-related transposons, we checked for a correlation between a number of identified elements and contig/scaffold N50 with Spearman’s rank correlation tests.

All confirmed LINEs (regardless of being autonomous or RT-only) and DDE/D-containing transposons were clustered at the nucleotide level using CD-HIT and following the 80–80 rule (same parameter set used for repeat library construction). Therefore, hereafter, we will refer to clusters as groups of TEs related by high nucleotide homology along their coding sequence to distinguish them from the canonical transposon families which ideally should take into consideration the elements along their entire length [7].

For LINE elements only, we additionally called “low-copy number clusters” clusters with less than 5 members and as “singleton cluster” sequences that did not fall in any cluster. For class II elements, we avoid such classification because non-autonomous members of a family can replicate through the genome parasitizing their autonomous counterparts. Moreover, while the presence of a complete ORF can give some first insight on which superfamilies/clades could have been more active in recent/mid times, on the other hand, it must be noted that this approach is not able to identify non-autonomous elements thus greatly underestimating the number of short Class II transposons.

### Tree-based classification of ORF-containing LINE elements

ORF-containing LINE elements were classified using a phylogenetic approach. We adopted the superfamily classification scheme proposed by [7] and the clade classification proposed by [29], as in [102], while we use the “type” term to refer to the RepeatMasker or Dfam classification schemes [103]. Starting from previously identified clusters (> 5 members), we extracted the amino acid sequence of the RT domain based on the coordinates of the RPSblast hits. RT segments were aligned with MAFFT v. 7.475 (*g-INS-i* strategy) and cleaned from columns with gaps in more than 50% of the sequences using TrimAl [104]. *Cons* from the EMBOSS package [105] was then used to build up a consensus sequence from the resulting alignment setting the parameter *plurality* to 3. RT consensus sequences were then aligned together with reference LINE sequences from [29] and a subset of LTR and LINE elements from the Repeatpeps library, using MAFFT and a *g-INS-i* strategy. Poorly aligned sequences were removed from the alignment using TrimaAl (*-resoverlap* 0.75 *-seqoverlap* 80). Because of the short RT domain, the deep divergence time of LINE superfamilies, and the consequently difficulties in identifying stable LINE phylogenies (e.g., [29, 30, 106]), we used a combination of neighbor-joining, unconstrained maximum likelihood (ML), and constrained ML tree inferences. Each topology was then statistically tested in a ML framework to produce a confident phylogeny useful for LINE classification. We performed (a) a neighbor-joining (NJ) clustering with Clearcut v. 1.0.9 [107], reshuffling the distance matrix and using a traditional neighbor-joining algorithm (*–shuffle* and *–neighbor* options, respectively); (b) 5 unconstrained maximum likelihood (ML) tree searches with IQtree v. 2.1.3 [108] and the corresponding best-fit evolutionary model identified by ModelFinder2 [109]; (c) 6 constrained ML tree searches forcing the full NJ topology (FullNJ constraint, one run); and (d) only the monophyly of LINEs superfamilies, as inferred by the NJ tree, with the exception of Jockey and I superfamilies which were constrained in a single, comprehensive monophyletic clade (SupFAM constraint, 5 runs). For the unconstrained and the SupFAM-constrained ML tree inferences (analyses b and d, respectively), nodal support was estimated with 1000 UltraFastBootstrap replicates [110]. All ML topologies were tested using the Kishino-Hasegawa test [111], Shimodaira-Hasegawa test [112], expected likelihood weights [113], and approximately unbiased (AU) test [114]. As an additional confirmation of our classification and to avoid the inclusion of Penelope-like elements, we (a) blasted each consensus RT (blastp; *E*-value < 1E − 5) against all protein sequences from the RepeatPeps library extracting the best-hit for each query sequence and (b) used the online implementation of RTClass1 [29] on a random subset of 111 RT sequences covering all identified clades. Low-copy numbers, singletons, and clusters removed by TrimAl were classified based on Blastp best-hit (*E*-value < 0.05) against tree-based classified clusters and the whole RepeatPeps library for competing purposes. For the low-copy clusters, one representative (i.e., the longest) sequence was used. For bivalve species, and excluding the poorly represented R2 superfamily, the correlation between the number of RT-containing LINEs and the number of clusters in each identified LINE clade was tested for each superfamily separately with Spearman’s rank correlation tests.

### Additional prediction of SINEs in a subset of selected species

To have a first insight into the SINE composition of bivalves, we selected 13 species (namely, *A. marissinca*, *C. sinensis*, *C. gigas*, *S. glomerata*, *T. granosa*, *S. broughtonii*, *M. coruscus*, *B. platifrons*, *S. constricta*, *S. grandis*, *P. maximus*, *M. yessoensis*, *M. nervosa*) representative of Venerida, Ostreida, Arcida, Mytilida, Adepedonta, Pectinida, and Unionida, to mine additional SINE candidates using SINE_Scan v1.1.1 [115]. This software collects and validates SINE candidates based on copy number across the genome, presence of target site duplications (TSDs), and trRNA-related heads. All representative elements were merged with consensus sequences classified as SINEs by RepeatModeler in the corresponding species-specific repeat library (see the “Class-level mollusc mobilome characterization using automatically generated TE sequence libraries” section) and subjected to manual validation and curation as described in the following section. After this process, curated consensus sequences were annotated using the RepeatClassifier utility from the RepeatModeler package.

### Manual curation of LINEs, SINEs, and DDE/D-related transposons

We selected a set of the previously found LINEs RT, SINEs, and DDE/D-containing clusters for manual refinement, following [27] guidelines. For LINEs, we selected all clusters with at least one autonomous element (i.e., encoding for an ORF with both RT and EN domains without interrupting stop codons) and five other sequences (both autonomous and/or RT-only) while for DDE/D elements, we required only the presence of at least five elements in the corresponding cluster. These criteria were chosen in order to prioritize the manual curation of sequences that likely possess one or more autonomous copies across the genome and thus could potentially be recently mobilized or mobilize their non-autonomous counterparts. Members of LINEs and DDE/D-related clusters were aligned at the nucleotide level using MAFFT (–*auto* strategy). CIAlign [116] was then used to remove insertions found in less than 50% of the sequences and to construct a nucleotide consensus sequence (*–remove-insertions* and *–make-consensus* option). At this set of LINEs and DDE/D preliminary consensus, we also added all the aforementioned SINEs, and all sequences were subjected to a “blast-extend-extract” process with a minimum required query coverage and identity of 70, extending each hit by 3 kb and extracting the top 25 hits for each query sequence and building up a preliminary consensus sequence using CIAlign. Resulting alignments were manually inspected to (i) identify structural features (e.g., microsatellites for LINEs and SINEs at the 3′ end 5′ truncations for LINEs, terminal inverted repeats, and superfamily-specific motifs for DDE/D elements), (ii) identify boundaries of the elements searching for TSDs whenever possible, (iii) identify domain signatures using the CDD web server, and (iv) correct and extend as long as possible the consensus sequence. Additionally, for SINE only, we also required (a) the presence of a detectable tRNA-related region at the 5′ ends and predicted with tRNAScan-SE (sequence source: mixed; score cutoff 0.01 [117]) and (b) the presence of a central domain and/or a tail region after the tRNA-related head. It must be noted that the presence of TSDs to confirm the boundaries of the element was only required for SINEs and class II superfamilies that exhibit them (thus excluding for example the SPY group from the PIF-Harbinger superfamily; see [118]), while for LINE elements, their presence was checked but not required because of difficulties in finding them due to frequent 5′ truncations. For LINEs, we instead rely on the distinctive decay of the alignment quality towards the 5′ end caused by 5′ truncations [27] curating each consensus until at least 3 sequences could confidently be aligned. Relationships between the number of curated families and the number of autonomous elements identified in each species was tested using Spearman’s rank correlation test.

### Genome annotation of LINEs and SINEs using manually curated libraries and phylogenetic inference of curated LINE families

After manual curation, we focused our analyses to the greatly understudied LINE complement. All LINE and SINE libraries were merged and CD-HIT-EST was used to remove redundant copies following the 80–80 rule. The merged non-redundant library was used in an additional RepeatMasker analysis in sensitive mode and increasing the minimum score to 400 from the default value of 225 (*-cutoff* 400), to remove low-scoring annotations. We tested for a correlation between genome coverage of LINEs and SINEs in the 12 selected species using Spearman’s rank correlation. For LINEs only, CpG-corrected Kimura distances of each copy from its consensus were calculated with the *calcDivFromAlign.pl* script from the RepeatMasker package. We define long-term survival families consensus that show both recent (< 5% divergence from the consensus) and ancient (> 30% divergence from the consensus) activity requiring a minimum of 30 copies in the recent and 5 in the ancient divergence bins. For this latest purpose, we applied a 3′ anchor-based counting method to reduce possible overestimations of the insertion number and spurious alignment between SINEs and their possible LINE counterparts. Briefly, we only count insertions that map to the first 50 nucleotides of the 3′ end of each consensus sequence and with a length of at least 100 bp based on aligned query and subject coordinates reported in the RepeatMasker out file.

Finally, from each LINE consensus sequence, we extracted the RT domain as previously described, and separately for each superfamily, we aligned all fragments and inferred a ML tree (MAFFT *g-INS-i* strategy; ModelFinder and IQ-TREE with 1000 Ultrafast Bootstrap replicates).

### Transcription potential of curated LINE families

To further test for activity potential of curated families in mature gonad tissues, we collected from NCBI paired-ends poly (A)-enriched RNA-seq data from mature male and female samples. Three biological replicates for each tissue were selected for *C. gigas* (SRR12564937, SRR12564938, SRR12564939, SRR12564936, SRR12564935, SRR12564940), *Chlamys farreri* (SRR5130887, SRR5130883, SRR5130863, SRR5130886, SRR5130875, SRR5130872), *M. yessoensis* (SRR9157572, SRR9157579, SRR9157580, SRR9157581, SRR9157582, SRR9157588), *Mercenaria mercenaria* (SRR10951876, SRR10951875, SRR10951874, SRR10951867, SRR10951866, SRR10951865), and *Sinonovacula constricta* (SRR9937011, SRR9937009, SRR9937008, SRR9937013, SRR9937012, SRR9937010). Raw reads were trimmed and deprived of adapters using bbduck from the bbmap package [119], requiring a minimum quality of 20 (*trimq* = *20*) and a minimum length of the reads after trimming of 75 (*minlen* = *75*). We decided to map all RNAseq reads only on 3′ anchored LINE insertions, as defined in the previous section, longer than 3000 bp and extracted with bedtools. These latest filters should ensure that reads originate from families that likely possess autonomous copies across the genome. To not discard multi mapping reads, we obtained a per-family raw count for each sample using TEtools [120] and bowtie2 [121] to align reads on extracted insertions. Raw counts were then normalized by the length of the corresponding family consensus sequences, and TPM values were calculated. Log2-transformed normalized counts were tested for a correlation with the number of previously identified 3′ anchored insertions with a minimum length of 100 bp for the corresponding family for each species, tissue, and biological replicate separately.

## Supplementary Information

**Additional file 1: Table S1.** Source and information of the 39 analyzed assemblies.

**Additional file 2: Fig. S1.** Schematic representation of the workflow used to create automatically generated repeat libraries and mined LINEs and DDE/D related transposons with ORF evidence (see “Mining and annotation of interspersed repeats” and “ORF-based annotation of RT containing LINEs and Class II DDE/D elements” sections).

**Additional file 3: Table S2.** Details about the number of de-novo sequences mined from software and used for automatic construction of species-specific repeats libraries (see “Mining and annotation of interspersed repeats” section).

**Additional file 4: Table S3.** Transposable element genomic content  of each transposon class using species-specific automatically generated TE sequence libraries (see “Mining and annotation of interspersed repeats” section).

**Additional file 5: Fig. S2.** (A) Copy number and (B) consensus length distribution of RepeatModeler “Unknown” consensus sequences (see “Mining and annotation of interspersed repeats” section).

**Additional file 6: Fig. S3.** Genome occupancy of each TE class in the 39 analyzed genomes using automatically generated TE libraries (see “Mining and annotation of interspersed repeats” section). Significant comparison are highlighted by asterisks (Pairwise Wilcoxon rank test with Bonferroni correction; * = *p* < 0.05, ** = ps < 0.01).

**Additional file 7: Fig. S4.** K-mean clustering obtained using 3 centers and based on the number of insertions for each transposon type as annotated by RepeatMasker using automatically generated TE libraries (see “Mining and annotation of interspersed repeats” section).

**Additional file 8: Table S4. **Results of ORF-based LINE annotation. N.Clusters=Number of cluster; N.RT=Number of RT-containing LINEs; RT.Clusters.Min5=Number of cluster with at least five members; RT.Seq.Clusters.Min5=Number of sequences contained in cluster with a size greater than 5; N.AE=number of putative autonomous LINEs. See “ORF-based annotation of RT containing LINEs and Class II DDE/D elements” section.

**Additional file 9: Fig. S5.** (A) Positive linear relationship between number of identified Reverse Transcriptase (RT) -containing LINE loci and number of autonomous elements (i.e., possessing both an RT and an Endonuclease domain) (Spearman’s rho=0.89, *p* < 0.01); (B) Positive linear relationship between scaffold N50 and number of identified Reverse Transcriptase containing LINE loci (Spearman’s rho=0.35, *p* < 0.05); (C) Positive linear relationship between scaffold N50 and number of identified LINE autonomous elements (Spearman’s rho=0.34, *p* < 0.05). See “ORF-based annotation of RT containing LINEs and Class II DDE/D elements” section.

**Additional file 10: Table S5.** Sequences included in phylogenetic analyses and used as reference to annotate molluscs LINEs (see “Tree-based classification of ORF-containing LINE elements” section). Reference = sequence obtained from Kapitov et al (2009) or from the RepeatPep library. RTClass = Subset of Mollusc LINEs annotated with RTClass1.

**Additional file 11: Fig. S6. **Superfamilies relationships obtained with Neighbor-Joining (A) and unconstrained Maximum Likelihood analyses (B). For the latter, only the run with the highest log-likelihood is shown (See Additional File 12: Table S6). All trees with nodal support values can be found in Additional File 29: Data S1. See “Tree-based classification of ORF-containing LINE elements” section.

**Additional file 12: Table S6.** Results of Maximum likelihood (ML) topology test between constrained and unconstrained tree searches (see “Tree-based classification of ORF-containing LINE elements” section). Tree with the highest log likelihood is highlighted in bold. FullNJ=ML tree obtained with a full constrain on the topology recovered by Neighbour-Joining; SupFAM=ML tree constrained only on the superfamilies relationships obtained by Neighbour-Joining. Plus signs denote accepted topologies by the respective topology test.

**Additional file 13: Fig. S7.** L1 superfamily subtree extracted from the SupFAM tree #2. Numbers on nodes represent UltraFast Bootstrap values. The outer-left annotation refers to the classification scheme proposed by RepBase and based on [29] while the outer-right based on the RepeatMasker “type” and obtained through blastp against the RepeatPep library. See “Tree-based classification of ORF-containing LINE elements” section.

**Additional file 14: Fig. S8.** RTE superfamily subtree extracted from the SupFAM tree #2. Numbers on nodes represent UltraFast Bootstrap values. The outer-left annotation refers to the classification scheme proposed by RepBase and based on [29] while the outer-right based on the RepeatMasker “type” and obtained through blastp against the RepeatPep library. See “Tree-based classification of ORF-containing LINE elements” section.

**Additional file 15: Fig. S9.** Jockey superfamily subtree extracted from the SupFAM tree #2. Numbers on nodes represent ultrafast bootstrap values. The outer-left annotation refers to the classification scheme proposed by RepBase and based on [29] while the outer-right based on the RepeatMasker “type” and obtained through blastp against the RepeatPep library. See “Tree-based classification of ORF-containing LINE elements” section.

**Additional file 16: Fig. S10.** I superfamily subtree extracted from the SupFAM tree #2. Numbers on nodes represent UltraFast Bootstrap values. The outer-left annotation refers to the classification scheme proposed by RepBase and based on [29] while the outer-right based on the RepeatMasker “type” and obtained through blastp against the RepeatPep library. See “Tree-based classification of ORF containing LINE elements” section.

**Additional file 17: Fig. S11.** R2 superfamily subtree extracted from the SupFAM tree #2. Numbers on nodes represent UltraFast Bootstrap values. The outer-left annotation refers to the classification scheme proposed by RepBase and based on [29] while the outer-right based on the RepeatMasker “type” and obtained through blastp against the RepeatPep library. See “Tree-based classification of ORF-containing LINE elements” section.

**Additional file 18: Fig. S12.** Number of RT-containing LINE clusters annotated in each analyzed genome and subdivided by clade following [29] and by RepeatMasker “type” classification in parenthesis. Clades are grouped by superfamily following [103] and the aforementioned SupFam tree #2. Note that the L2-2 clade includes Crack, Daphne, L2A and L2B elements. See “Tree-based classification of ORF-containing LINE elements” section.

**Additional file 19: Fig. S13.** Scatterplot of number of RT-containing LINEs and clusters for L1, Jockey, RTE and I superfamilies. The R2 superfamily was not included because of the low number of data points. Each point represents a clade/type. See “Tree-based classification of ORF-containing LINE elements” section.

**Additional file 20: Fig. S14.** Number of autonomous elements annotated in each analyzed genome and subdivided by clade following [29] and by RepeatMasker “type” classification in parenthesis. Clades are grouped by superfamily following [103] and the aforementioned SupFam tree #2. Note that the L2-2 clade includes Crack, Daphne, L2A and L2B elements. See “ORF-based annotation of RT containing LINEs and Class II DDE/D elements” and “Tree-based classification of ORF-containing LINE elements” sections.

**Additional file 21: Fig. S15.** Length distribution of manually curated LINE families. Each bar represents an element and colors denote different LINE superfamilies/types. See “Manual curation of LINEs, SINEs, and DDE/D-related transposons” section.

**Additional file 22: Fig. S16.** Length distribution of manually curated DDE/D transposon families. Each bar represents an element and colors denote different superfamilies. See “Manual curation of LINEs, SINEs, and DDE/D-related transposons” section.

**Additional file 23: Fig. S17.** Phylogenetic tree of curated bivalves LINEs RTE families. Numbers on nodes represent UltraFast Bootstrap values. See “Genome annotation of LINEs and SINEs using manually curated libraries and phylogenetic inference of curated LINE families” section.

**Additional file 24: Fig. S18.** Phylogenetic tree of curated bivalves LINEs I families. Numbers on nodes represent UltraFast Bootstrap values. See “Genome annotation of LINEs and SINEs using manually curated libraries and phylogenetic inference of curated LINE families” section.

**Additional file 25: Fig. S19.** Phylogenetic tree of curated bivalves LINEs L1 families. Numbers on nodes represent UltraFast Bootstrap values. See “Genome annotation of LINEs and SINEs using manually curated libraries and phylogenetic inference of curated LINE families” section.

**Additional file 26: Fig. S20.** Phylogenetic tree of curated bivalves LINEs Jockey families. Numbers on nodes represent UltraFast Bootstrap values. See “Genome annotation of LINEs and SINEs using manually curated libraries and phylogenetic inference of curated LINE families” section.

**Additional file 27: Table S7.** Annotation of RT-containing LINEs included in phylogenetic analyses. Superfamily and clade classification are based on reference sequences extracted from RepBase and follow [7] and [29], RM Type=Classification based on best-hit blastp results against the full RepeatPep library extracted from the RepeatMasker package; RT members=Number of RT-containing LINEs included in each cluster.

**Additional file 28: Table S8.** Conserved Domain Database identifier used to search for Reverse Transcriptase and Endonuclease signatures in extracted open reading frames.

**Additional file 29: Data S1.** LINE phylogenetic trees.

**Additional file 30: Data S2.** Multi Sequence Alignment used to generate all phylogenetic trees.

**Additional file 31: Data S3.** Library of curated LINE families. All sequences have been classified following a RepeatMasker formatting style.

**Additional file 32: Data S4.** Library of curated SINE families. All sequences have been classified following a RepeatMasker formatting style.

**Additional file 33: Data S5.** Library of curated Class II DDE/D-related families. These families correspond to full length elements. All sequences have been classified following a RepeatMasker formatting style.

## Data Availability

All data generated or analyzed during this study are included in this published article, its supplementary information files, and publicly available repositories. Phylogenetic trees can be found in Additional file 29: Data S1 together with the multiple sequence alignment used to generate them in Additional file 30: Data S2. Manually curated families can be found in Additional files 31, 32, and 33 with a RepeatMasker formatted style as well as in the GitHub repository (https://github.com/CompBio-BO/Bivalvia_TEs) and in DFAM under Creative Commons CC0 1.0 public domain license. All supplementary data have been also deposited in a figshare database under the https://doi.org/10.6084/m9.figshare.22188280.v1 [122]. Scripts used to automatically generate the species-specific repeat libraries and to extract LINEs and DDE/D-related ORFs can be found in GitHub (https://github.com/jacopoM28/EvoTEs_BiV) and in Zenodo under the https://doi.org/10.5281/zenodo.7944844 [123].

## Abbreviations

TE: Transposable elements
LINEs: Long interspersed nuclear elements
RT: Reverse transcriptase
EN: Endonuclease
SINEs: Short interspersed nuclear elements
LTR: Long terminal repeat
TIR: Terminal inverted repeats
MITEs: Miniature inverted-repeats transposable elements
RC: Rolling circle
MULE: Mutator-like elements
