# Supplementary material for: Multiple and diversified transposon lineages contribute to early and recent bivalve genome evolution
Source: BMC Biol. 2023 Jun 26;21:145. doi: 10.1186/s12915-023-01632-z (PMC10294476; doi:10.1186/s12915-023-01632-z)
Supplement: Supplementary file 1 — Additional file 1: Table S1. Source and information of the 39 analyzed assemblies. [file 12915_2023_1632_MOESM1_ESM.docx]

**Tab. S1.** Source and information of the 39 analyzed assemblies.

| **Species** | **Genome source** | **Assembly level** | **Assembly length (bp)** | **Sequencing coverage** | **Sequencing technology** | **Contig N50 (bp)** | **Scaffold N50 (bp)** | **Taxonomy** |
| --- | --- | --- | --- | --- | --- | --- | --- | --- |
| *Helobdella robusta* | NCBI; GCA_000326865.1 | scaffold | 235,376,169 | 7.9X | Sanger | 52,195 | 3,060,193 | Annelida |
| *Dinophilus gyrociliatus* | NCBI; GCA_904063045.1 | Scaffold | 77,897,245 | 73X | PacBio | 979,161 | 2,241,898 | Annelida |
| *Capitella teleta* | NCBI; GCA_000328365.1 | Scaffold | 333,283,208 | 7.9X | Sanger | 21,930 | 188,402 | Annelida |
| *Acanthopleura granulata* | NCBI; GCA_016165875.1 | Scaffold | 606,536,932 | 40X | Oxford Nanopore + Illumina | 1,098,986 | 23,921,462 | Polyplacophora |
| *Octopus sinensis* | NCBI; GCF_006345805.1 | Chromosome | 2,719,151,902 | 285X | PacBio + Illumina | 490,217 | 105,892,736 | Cephalopoda |
| *Octopus bimaculoides* | NCBI; GCF_001194135.1 | Scaffold | 2,338,188,782 | 92X | Illumina | 5,532 | 475,182 | Cephalopoda |
| *Lottia gigante* | NCBI; GCF_000327385.1 | Scaffold | 359,505,668 | 8.87X | Sanger | 96,027 | 1,870,055 | Gastropoda |
| *Crysomallon squamiferum* | Dryad; https://doi.org/10.5061/dryad.24053dn | Chromosome | 404,615,235 | NA | Oxford Nanoporer + Illumina | 1,880,000 | 30,197,626 | Gastropoda |
| *Pomacea canaliculata* | NCBI; GCF_003073045.1 | Chromosome | 440,159,624 | 60X | PacBio + Illumina | 1,072,857 | 31,531,291 | Gastropoda |
| *Biomphalaria glabrata* | NCBI; GCF_000457365.1 | Scaffold | 916,388,084 | 27.5X | 454 FLX Titanium | 7,298 | 48,059 | Gastropoda |
| *Acanthina immaculata* | NCBI; GCA_009760885.1 | Chromosome | 1,653,153,977 | 125X | PacBio + Illumina | 3,802,429 | 56,367,627 | Gastropoda |
| *Potamilus streckersoni* | NCBI; GCA_016746295.1 | Scaffold | 1,776,755,686 | 100X | PacBio + 10X Genomics | 2,032,685 | 2,051,244 | Bivalvia - Paleoheterodonta - Unionida |
| *Megalonaias nervosa* | NCBI; GCA_016617855.1 | Scaffold | 2,365,218,322 | 40X | Oxford Nanopore + Illumina | 50,186 | 50,649 | Bivalvia - Paleoheterodonta - Unionida |
| *Solen grandis* | NCBI; GCA_021229015.1 | Chromosome | 1,324,491,672 | 10X | Illumina | 50,000 | 67,678,117 | Bivalvia - Imparidentia - Adepedonta |
| *Sinonovacula constricta* | NCBI; GCA_007844125.1 | Chromosome | 1,220,848,272 | 561X | PacBio + Illumina | 976,936 | 65,929,677 | Bivalvia - Imparidentia - Adepedonta |
| *Dreissena rostriformis* | Phaidra; https://phaidra.univie.ac.at/view/o:980132 | Scaffold | 1,241,502,953 | 92X | Illumina | / | 131.400 | Bivalvia - Imparidentia - Myda |
| *Archivesica marissinica* | NCBI; GCA_014843695.1 | Chromosome | 1,544,610,695 | 168X | PacBio + Illumina | 79,144 | 74,312,544 | Bivalvia - Imparidentia - Venerida |
| *Cyclina sinensis* | NCBI; GCA_012932295.1 | Scaffold | 903,119,975 | 447X | PacBio + Illumina | 2,587,078 | 46,470,132 | Bivalvia - Imparidentia - Venerida |
| *Mercenaria mercenaria* | NCBI; GCA_014805675.1 | Chromosome | 1,788,352,626 | 555X | PacBio + Illumina | 1,773,430 | 91,379,220 | Bivalvia - Imparidentia - Venerida |
| *Ruditapes philippinarum* | NCBI: GCA_026571515.1 | Contig | 1,408,186,117 | 25X | PacBio + Illumina | 183,074 | / | Bivalvia - Imparidentia - Venerida |
| *Anadara kagoshimensis* | NCBI; GCA_021292105.1 | Chromosome | 1,115,236,308 | 103X | PacBio | 1,935,399 | 60,635,260 | Bivalvia - Pteriomorpha - Arcida |
| *Scapharca broughtonii* | GigaDB; [h](http://dx.doi.org/10.5524/100607)ttp://dx.doi.org/10.5524/100607 | Chromosome | 884,566,040 | NA | PacBio + Nanopore + Illumina | 1,797,717 | 44,995,656 | Bivalvia - Pteriomorphia - Arcida |
| *Tegillarca granosa* | NCBI; GCA_013375625.1 | Scaffold | 797,648,519 | 200X | PacBio | 605,873 | 42,616,908 | Bivalvia - Pteriomorphia - Arcida |
| *Myzuhopecten yessoensis* | NCBI; GCF_002113885.1 | Scaffold | 987,588,634 | 297X | Illumina | 65,014 | 803,631 | Bivalvia - Pteriomorphia - Pectinida |
| *Chlamys farreri* | MolluscDB; http://mgbase.qnlm.ac/home | Scaffold | 816,498,441 | 382X | Illumina | 21,500 | 555,171 | Bivalvia - Pteriomorphia - Pectinida |
| *Pecten maximus* | NCBI; GCF_902652985.1 | Chromosome | 918,306,378 | 54X | PacBio + 10X Genomics | 1,258,799 | 44,824,366 | Bivalvia - Pteriomorphia - Pectinida |
| *Argopecten irradians concentricus* | Dryad; https://datadryad.org/stash/dataset/doi:10.5061/dryad.hdr7sqvdr | Scaffold | 874,821,818 | 382X | Illumina | 63,730 | 1,246,717 | Bivalvia - Pteriomorphia - Pectinida |
| *Argopecten purpuratus* | MolluscDB  http://mgbase.qnlm.ac/home | Scaffold | 724,780,576 | 148X | PacBio + Illumina | 81,100 | 1,022,003 | Bivalvia - Pteriomorpha - Pectinidae |
| *Pinctada fucata* | GigaDB;  http://gigadb.org/dataset/100240 | Chromosome | 991,016,007 | 234X | Illumina | 21,000 | 59,022,695 | Bivalvia - Pteriomorphia - Ostreida |
| *Saccostrea glomerata* | dbSROG; http://soft.bioinfo-minzhao.org/srog/# | Scaffold | 788,118,542 | 300X | Illumina | 39,800 | 804,232 | Bivalvia - Pteriomorphia - Ostreida |
| *Crassostrea virginica* | NCBI; GCF_002022765.2 | Chromosome | 684,741,128 | 87X | PacBio | 1,971,208 | 75,944,018 | Bivalvia - Pteriomorphia - Ostreida |
| *Crassostrea ariakensis* | NCBI; GCA_020567875.1 | Chromosome | 613,913,980 | 299X | Oxford Nanopore | 4,726,206 | 62,260,571 | Bivalvia - Pteriomorphia - Ostreida |
| *Crassostrea gigas* | NCBI; GCF_902806645.1 | Chromosome | 647,887,097 | 70X | PacBio + Illumina | 1,564,469 | 58,462,999 | Bivalvia - Pteriomorphia - Ostreida |
| *Mytilus coruscus* | NCBI; GCA_017311375.1 | Chromosome | 1,566,529,938 | 160X | Oxford Nanopore | 1,481,111 | 99,542,347 | Bivalvia Pteriomorphia - Mytilida |
| *Mytilus edulis* | NCBI; GCA_019925275.1 | Chromosome | 1,651,313,236 | 196X | PacBio | 490,737 | 116,503,180 | Bivalvia Pteriomorphia - Mytilida |
| *Limnoperna fortunei* | GigaDB; http://gigadb.org/dataset/100386 | Scaffold | 1,673,125,894 | 60X | PacBio + Illumina | 32,203 | 312,020 | Bivalvia Pteriomorphia - Mytilida |
| *Modiolus philippinarum* | Dryad; https://datadryad.org/stash/dataset/doi:10.5061/dryad.h9942 | Scaffold | 2,629,649,654 | 209X | Illumina | 18,389 | 100,161 | Bivalvia Pteriomorphia - Mytilida |
| *Bathymodiolus platifrons* | Dryad; https://datadryad.org/stash/dataset/doi:10.5061/dryad.h9942 | Scaffold | 1,659,280,971 | 319X | Illumina | 12,602 | 343,373 | Bivalvia Pteriomorphia - Mytilida |
