# Supplementary figures and images for: Multiple and diversified transposon lineages contribute to early and recent bivalve genome evolution
Source: BMC Biol. 2023 Jun 26;21:145. doi: 10.1186/s12915-023-01632-z (PMC10294476; doi:10.1186/s12915-023-01632-z)

Figure S1

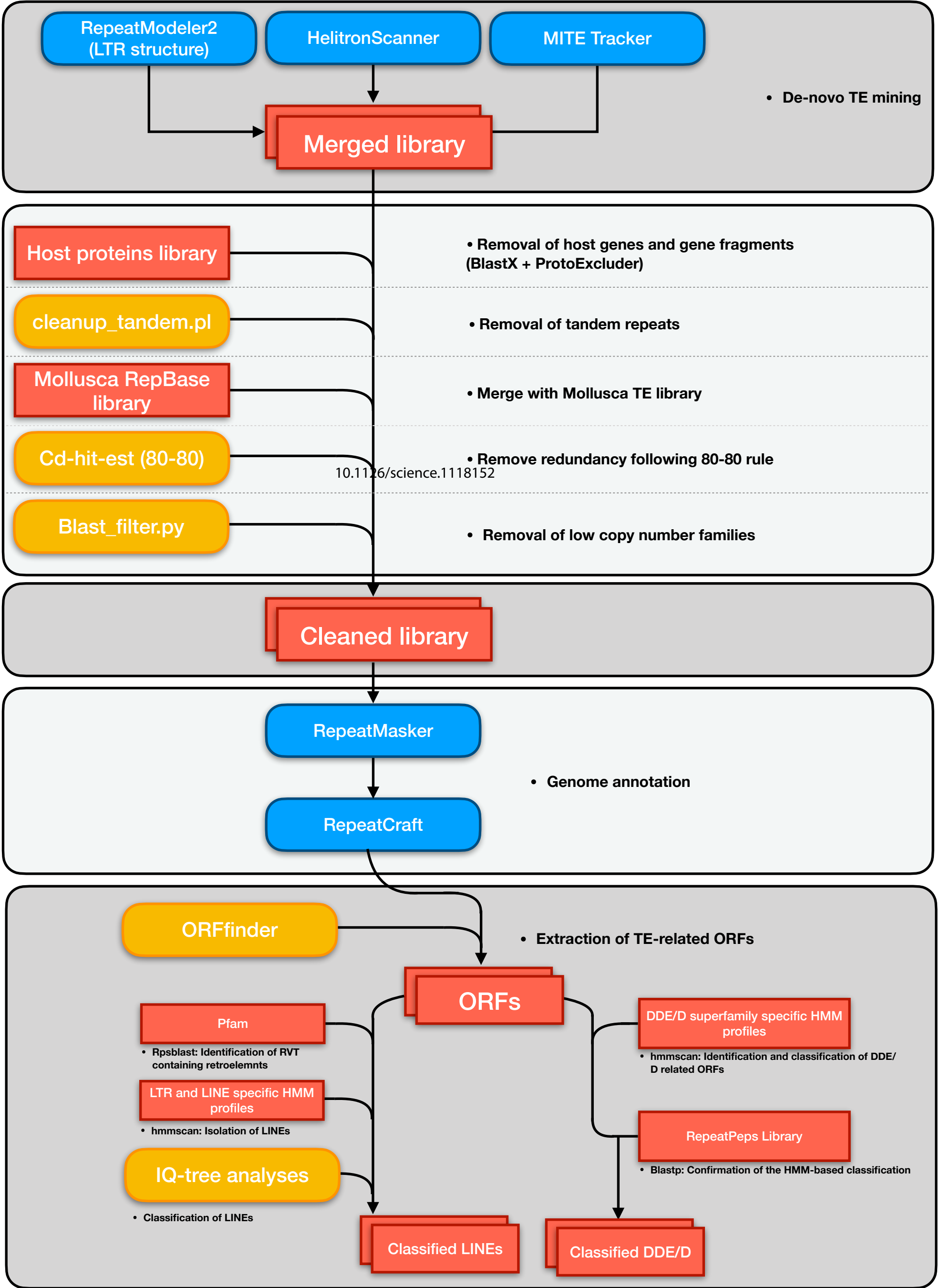

Supplement: Supplementary file 2 — Additional file 2: Fig. S1. Schematic representation of the workflow used to create automatically generated repeat libraries and mined LINEs and DDE/D related transposons with ORF evidence (see “Mining and annotation of interspersed repeats” and “ORF-based annotation of RT containing LINEs and Class II DDE/D elements” sections). [file 12915_2023_1632_MOESM2_ESM.pdf]

# Figure S2

## A

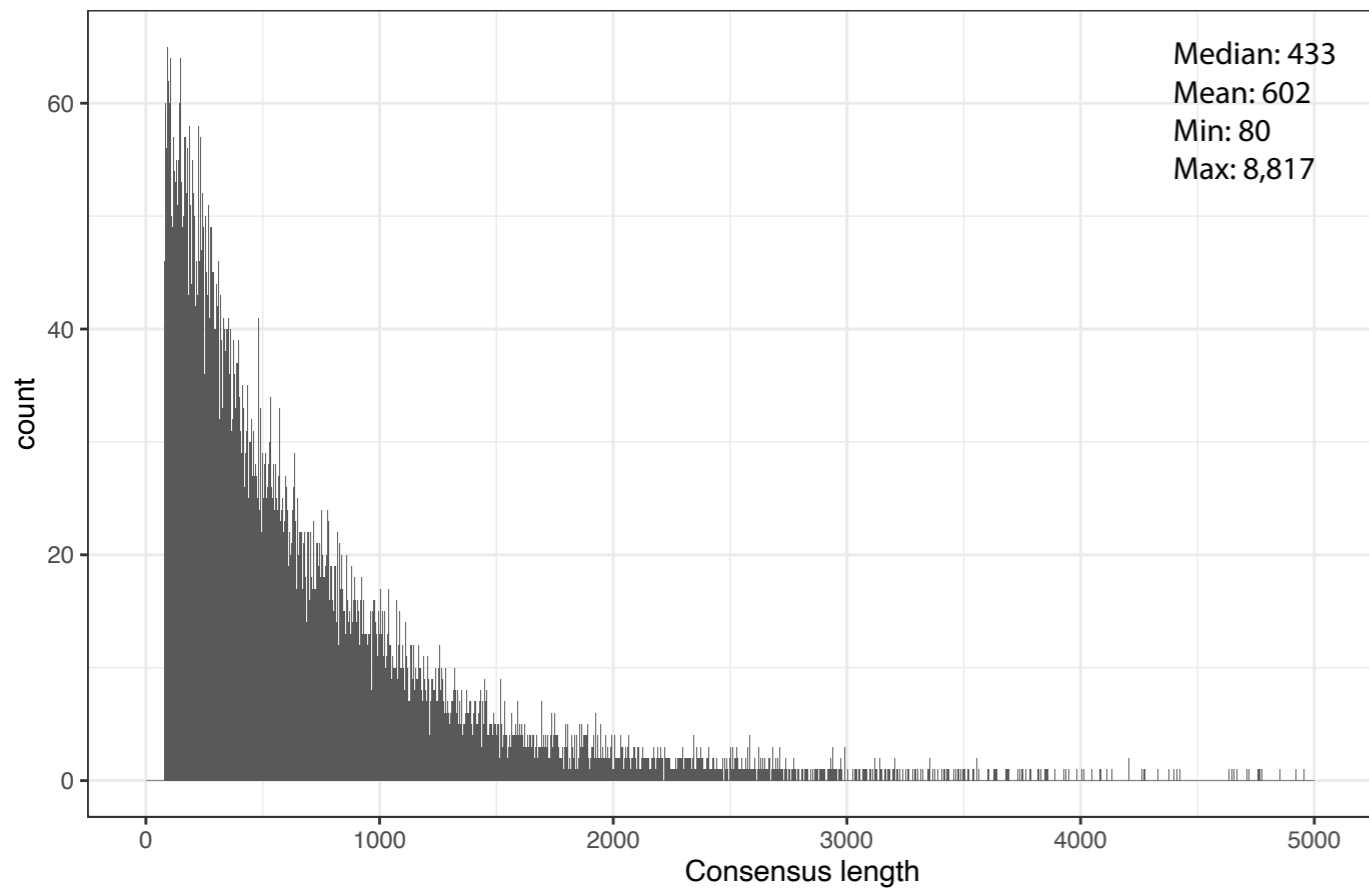

## B

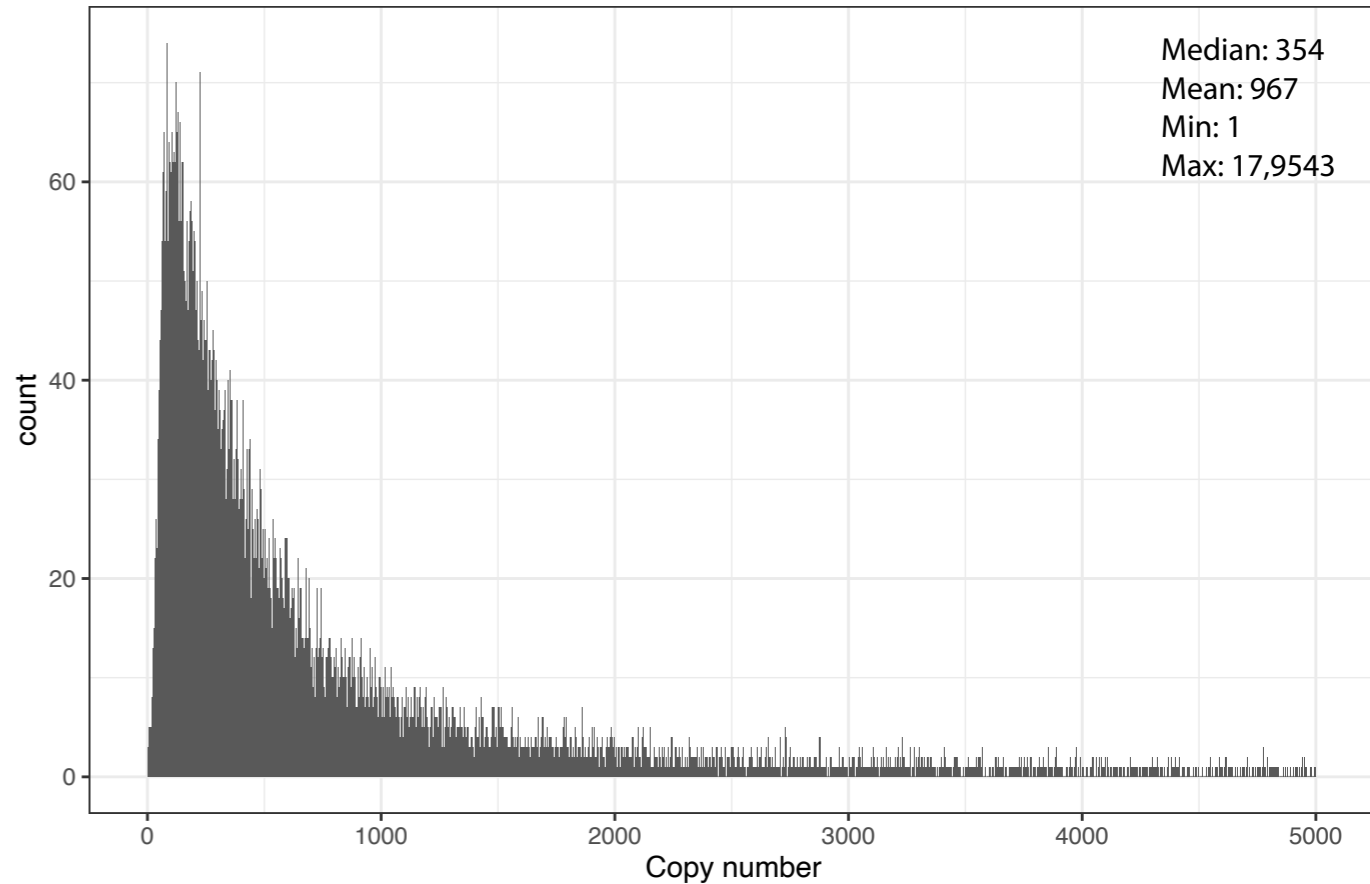

Supplement: Supplementary file 5 — Additional file 5: Fig. S2. (A) Copy number and (B) consensus length distribution of RepeatModeler “Unknown” consensus sequences (see “Mining and annotation of interspersed repeats” section). [file 12915_2023_1632_MOESM5_ESM.pdf]

# Figure S3

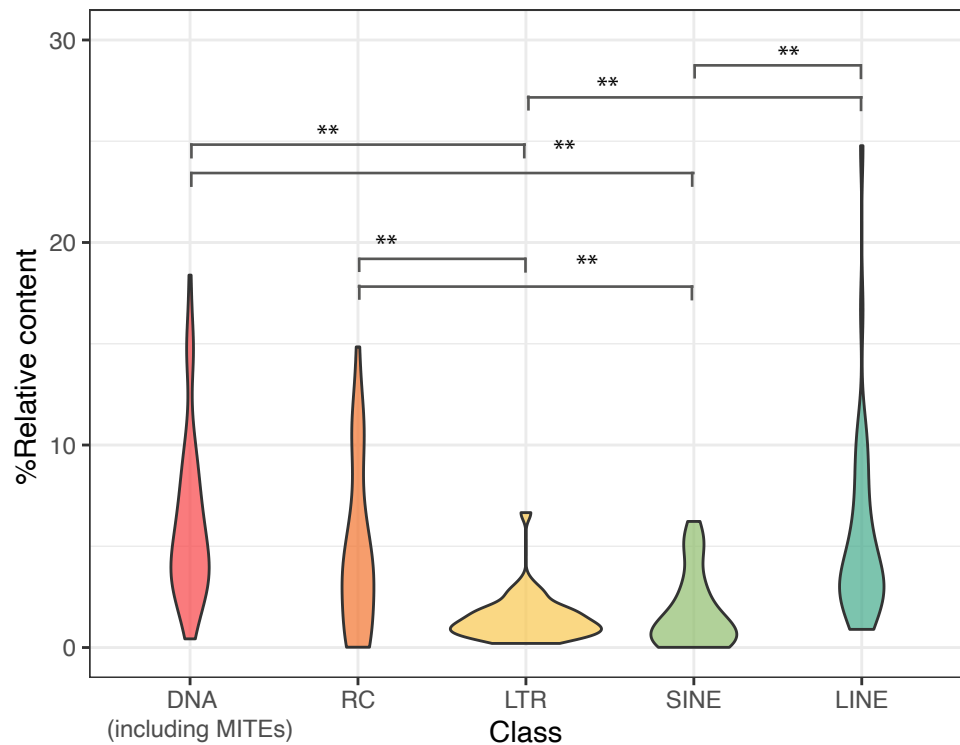

Supplement: Supplementary file 6 — Additional file 6: Fig. S3. Genome occupancy of each TE class in the 39 analyzed genomes using automatically generated TE libraries (see “Mining and annotation of interspersed repeats” section). Significant comparison are highlighted by asterisks (Pairwise Wilcoxon rank test with Bonferroni correction; * = p < 0.05, ** = ps < 0.01). [file 12915_2023_1632_MOESM6_ESM.pdf]

# Figure S4

Cluster plot

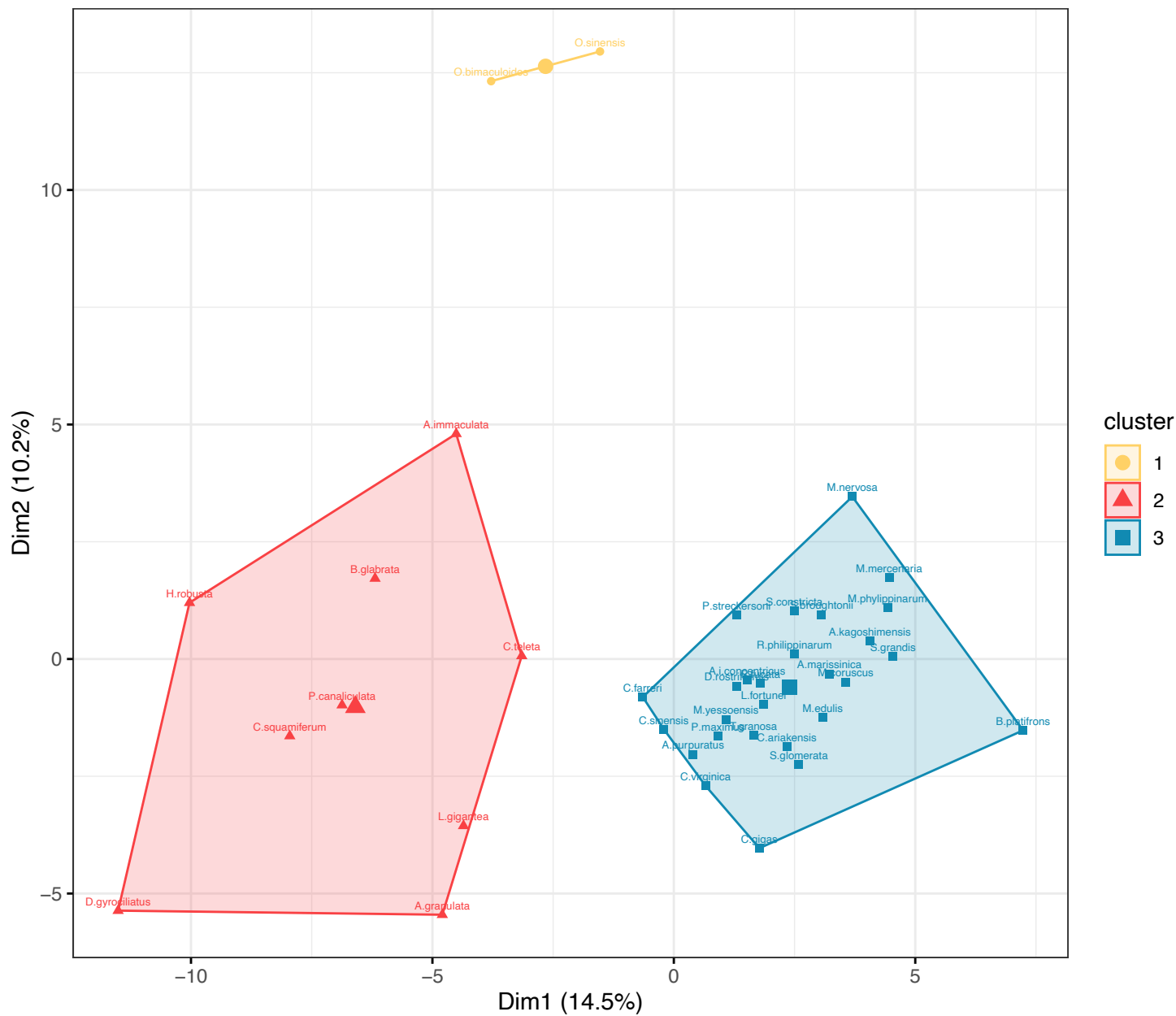

Supplement: Supplementary file 7 — Additional file 7: Fig. S4. K-mean clustering obtained using 3 centers and based on the number of insertions for each transposon type as annotated by RepeatMasker using automatically generated TE libraries (see “Mining and annotation of interspersed repeats” section). [file 12915_2023_1632_MOESM7_ESM.pdf]

# Figure S5

## A

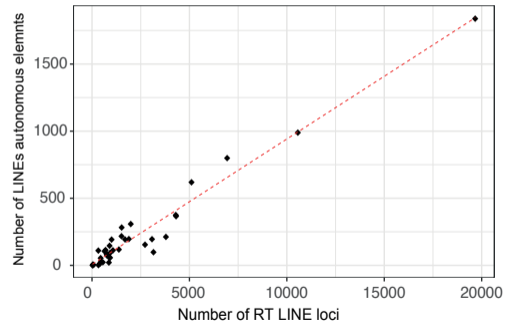

## B

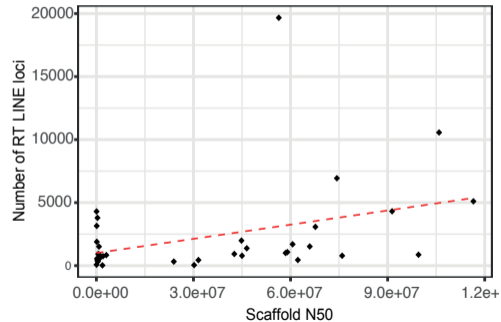

## C

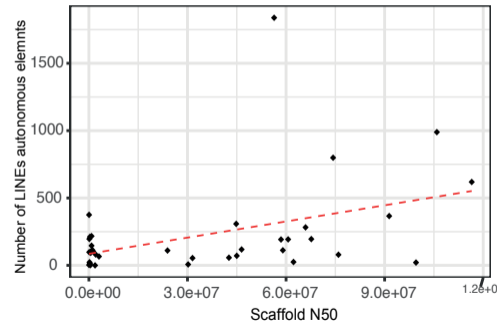

Supplement: Supplementary file 9 — Additional file 9: Fig. S5. (A) Positive linear relationship between number of identified Reverse Transcriptase (RT) -containing LINE loci and number of autonomous elements (i.e., possessing both an RT and an Endonuclease domain) (Spearman’s rho=0.89, p < 0.01); (B) Positive linear relationship between scaffold N50 and number of identified Reverse Transcriptase containing LINE loci (Spearman’s rho=0.35, p < 0.05); (C) Positive linear relationship between scaffold N50 and number of identified LINE autonomous elements (Spearman’s rho=0.34, p < 0.05). See “ORF-based annotation of RT containing LINEs and Class II DDE/D elements” section. [file 12915_2023_1632_MOESM9_ESM.pdf]

# Figure S6

NJ Topology

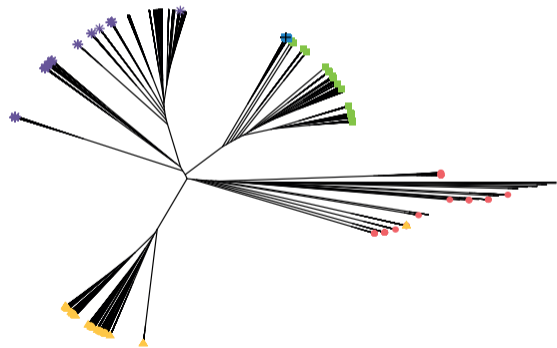

Superfamily

- I
- ▲ Jockey
- L1
- + LTR
- ▣ R2
- \* RTE

ML unconstrained

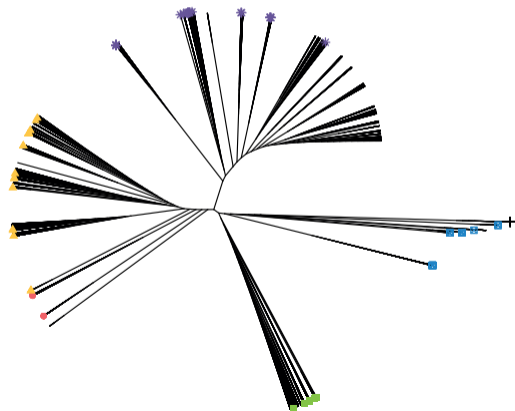

Supplement: Supplementary file 11 — Additional file 11: Fig. S6. Superfamilies relationships obtained with Neighbor-Joining (A) and unconstrained Maximum Likelihood analyses (B). For the latter, only the run with the highest log-likelihood is shown (See Additional File 12: Table S6). All trees with nodal support values can be found in Additional File 29: Data S1. See “Tree-based classification of ORF-containing LINE elements” section. [file 12915_2023_1632_MOESM11_ESM.pdf]

Figure S7

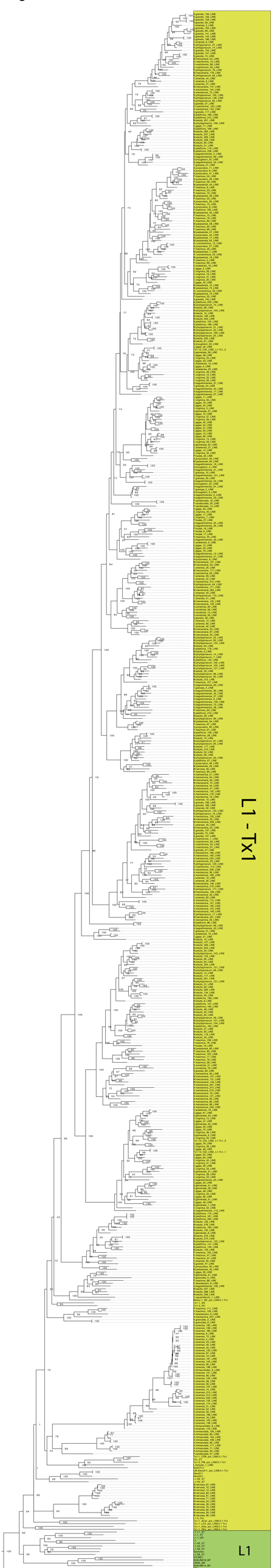

Supplement: Supplementary file 13 — Additional file 13: Fig. S7. L1 superfamily subtree extracted from the SupFAM tree #2. Numbers on nodes represent UltraFast Bootstrap values. The outer-left annotation refers to the classification scheme proposed by RepBase and based on [29] while the outer-right based on the RepeatMasker “type” and obtained through blastp against the RepeatPep library. See “Tree-based classification of ORF-containing LINE elements” section. [file 12915_2023_1632_MOESM13_ESM.pdf]

Figure S10

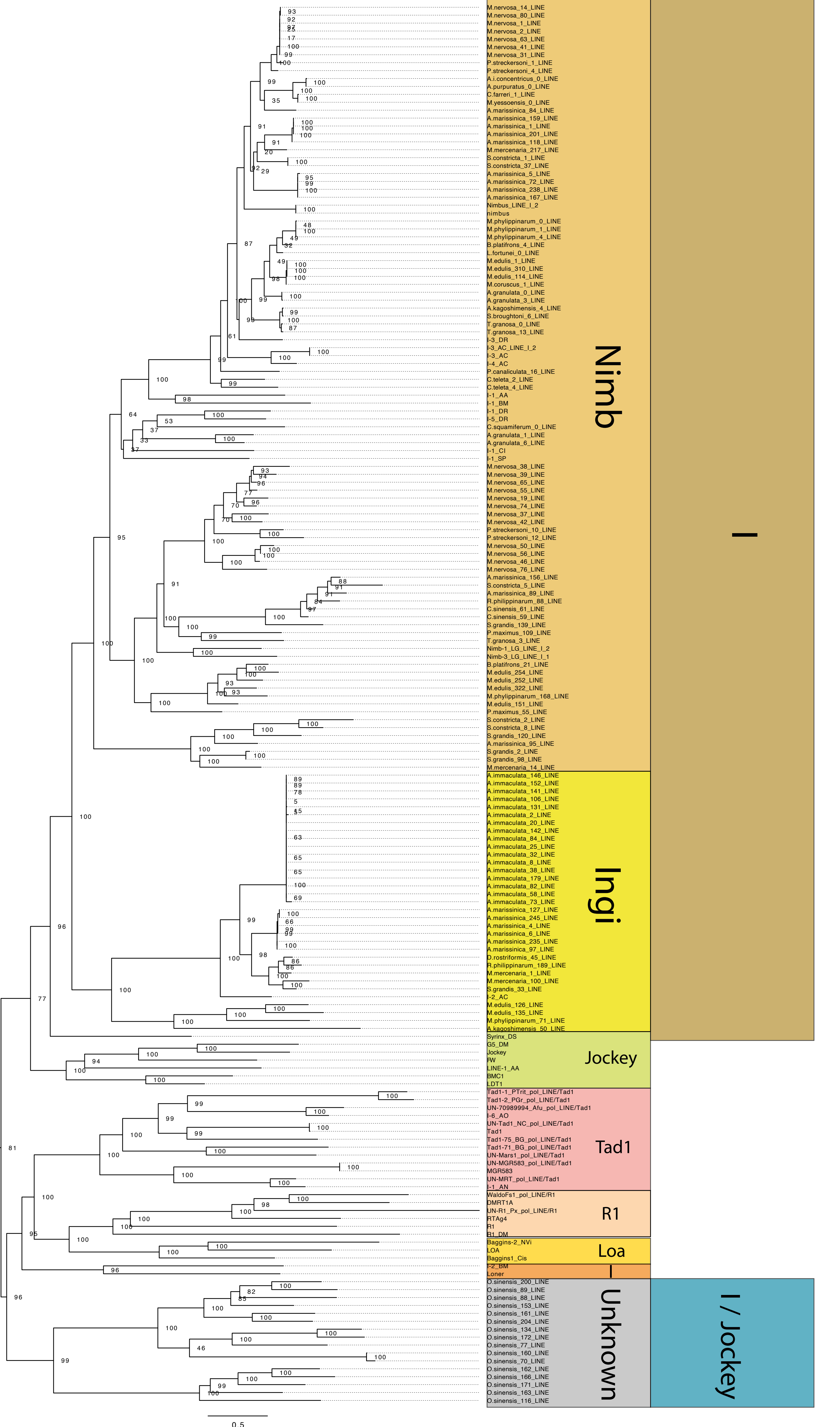

Supplement: Supplementary file 16 — Additional file 16: Fig. S10. I superfamily subtree extracted from the SupFAM tree #2. Numbers on nodes represent UltraFast Bootstrap values. The outer-left annotation refers to the classification scheme proposed by RepBase and based on [29] while the outer-right based on the RepeatMasker “type” and obtained through blastp against the RepeatPep library. See “Tree-based classification of ORF containing LINE elements” section. [file 12915_2023_1632_MOESM16_ESM.pdf]

Figure S11

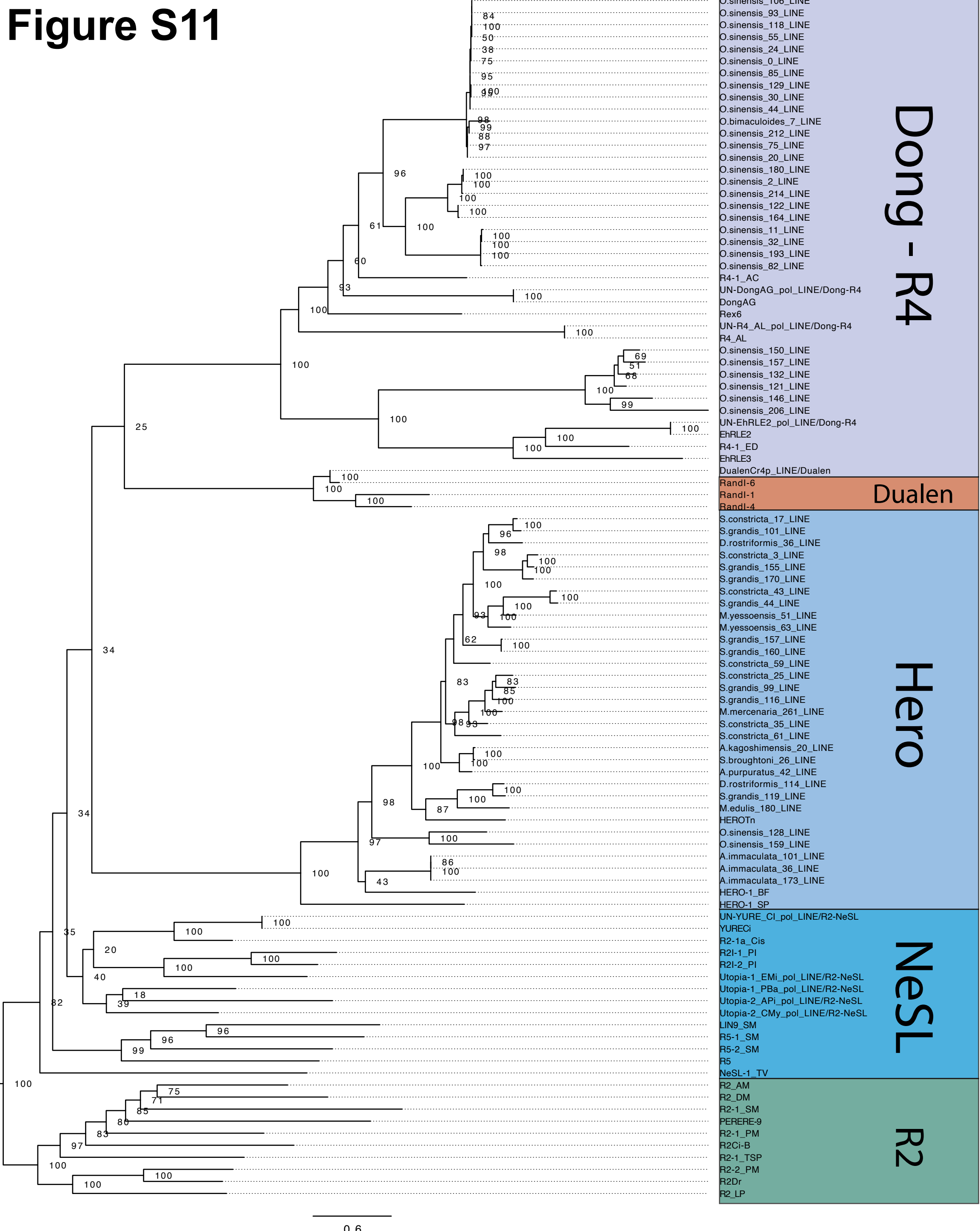

Supplement: Supplementary file 17 — Additional file 17: Fig. S11. R2 superfamily subtree extracted from the SupFAM tree #2. Numbers on nodes represent UltraFast Bootstrap values. The outer-left annotation refers to the classification scheme proposed by RepBase and based on [29] while the outer-right based on the RepeatMasker “type” and obtained through blastp against the RepeatPep library. See “Tree-based classification of ORF-containing LINE elements” section. [file 12915_2023_1632_MOESM17_ESM.pdf]

Figure S12

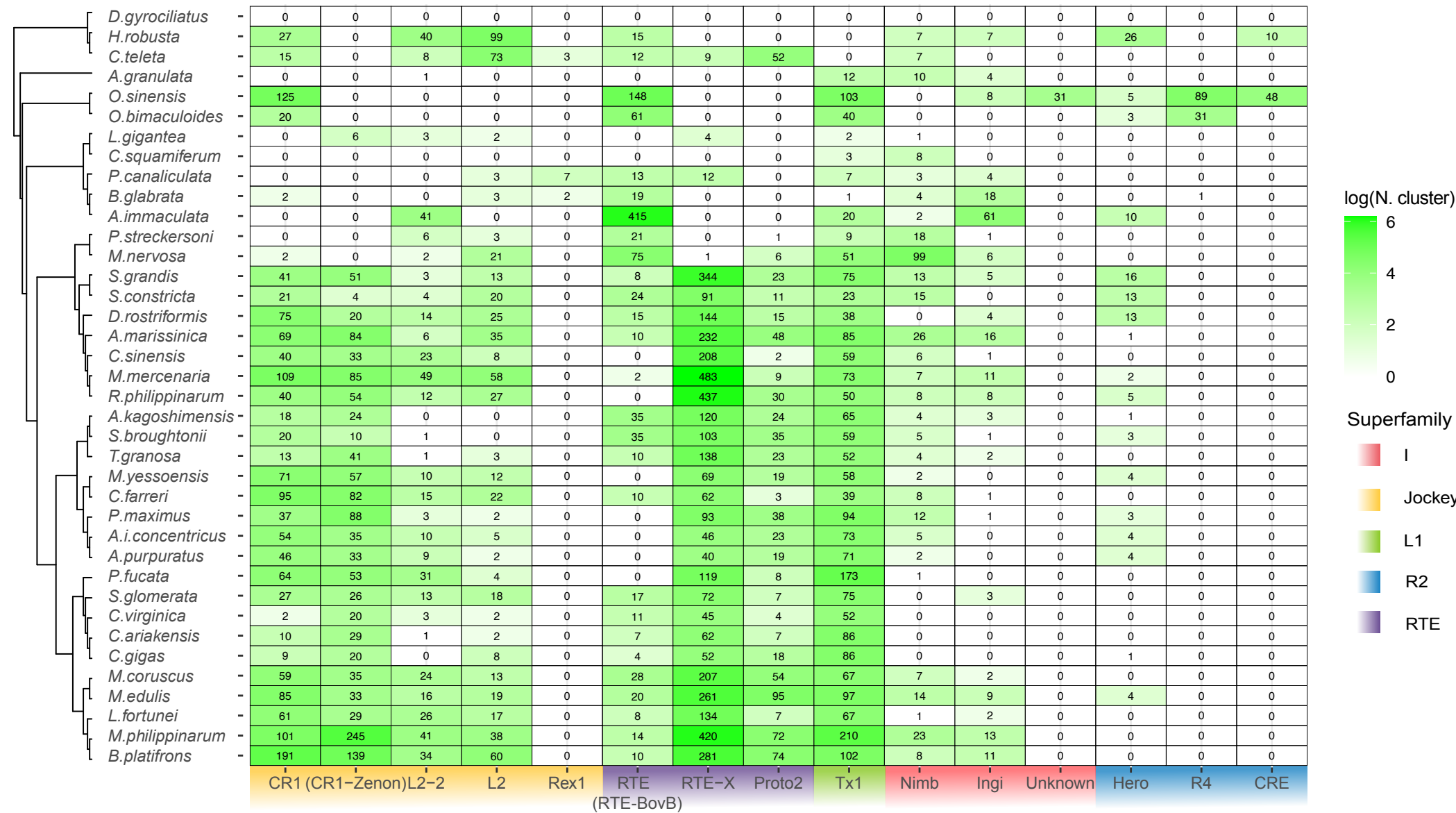

Supplement: Supplementary file 18 — Additional file 18: Fig. S12. Number of RT-containing LINE clusters annotated in each analyzed genome and subdivided by clade following [29] and by RepeatMasker “type” classification in parenthesis. Clades are grouped by superfamily following [103] and the aforementioned SupFam tree #2. Note that the L2-2 clade includes Crack, Daphne, L2A and L2B elements. See “Tree-based classification of ORF-containing LINE elements” section. [file 12915_2023_1632_MOESM18_ESM.pdf]

# Figure S13

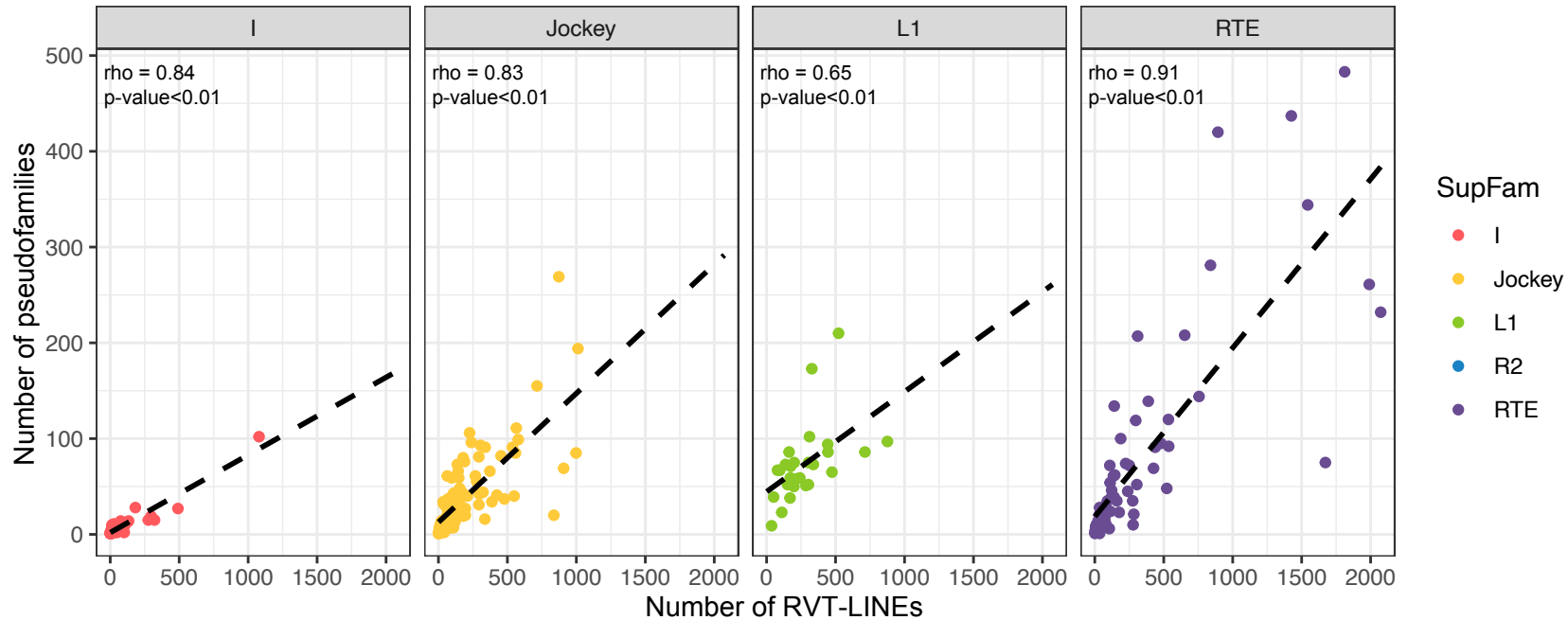

Supplement: Supplementary file 19 — Additional file 19: Fig. S13. Scatterplot of number of RT-containing LINEs and clusters for L1, Jockey, RTE and I superfamilies. The R2 superfamily was not included because of the low number of data points. Each point represents a clade/type. See “Tree-based classification of ORF-containing LINE elements” section. [file 12915_2023_1632_MOESM19_ESM.pdf]

Figure S14

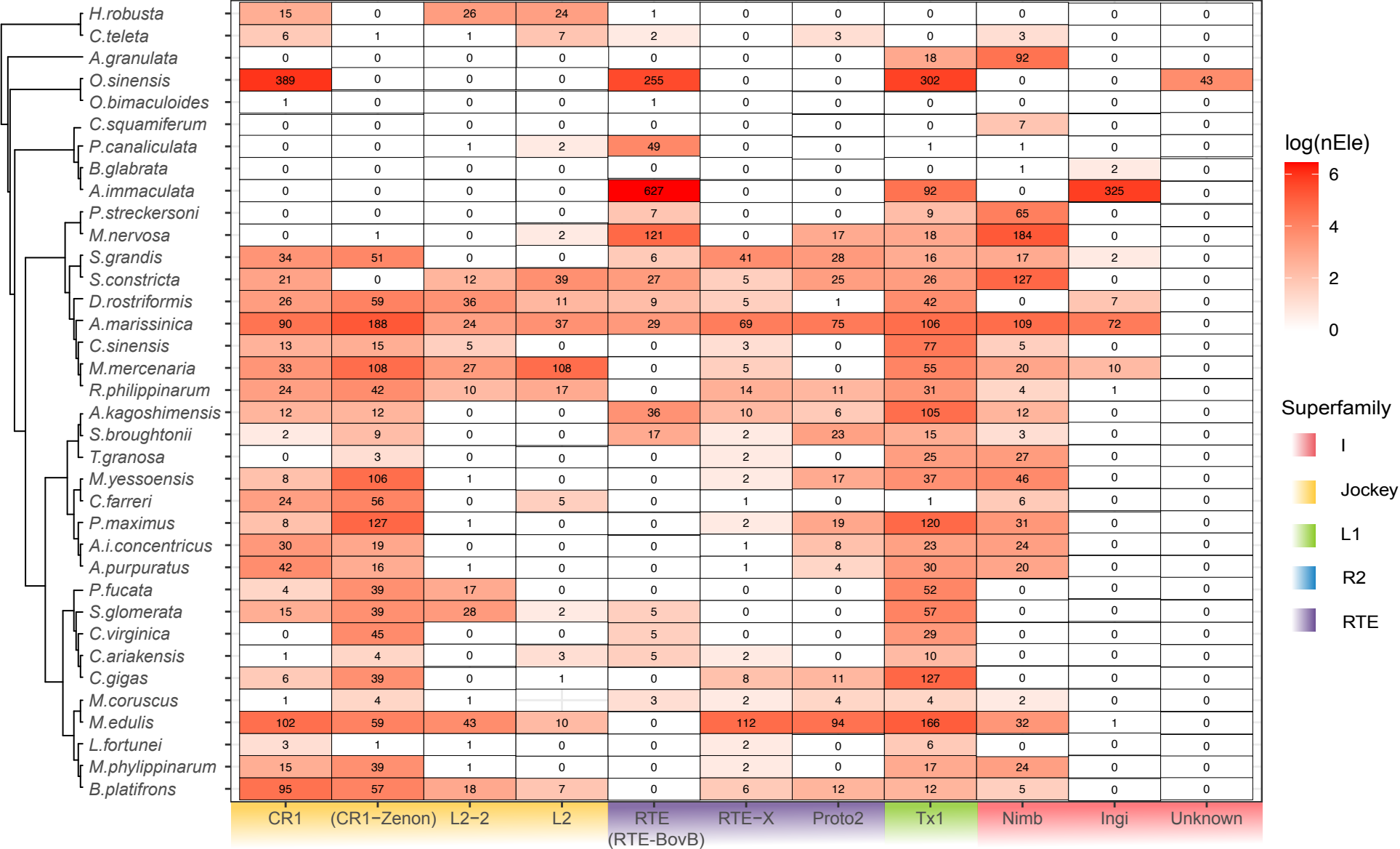

Supplement: Supplementary file 20 — Additional file 20: Fig. S14. Number of autonomous elements annotated in each analyzed genome and subdivided by clade following [29] and by RepeatMasker “type” classification in parenthesis. Clades are grouped by superfamily following [103] and the aforementioned SupFam tree #2. Note that the L2-2 clade includes Crack, Daphne, L2A and L2B elements. See “ORF-based annotation of RT containing LINEs and Class II DDE/D elements” and “Tree-based classification of ORF-containing LINE elements” sections. [file 12915_2023_1632_MOESM20_ESM.pdf]

**Figure S15**

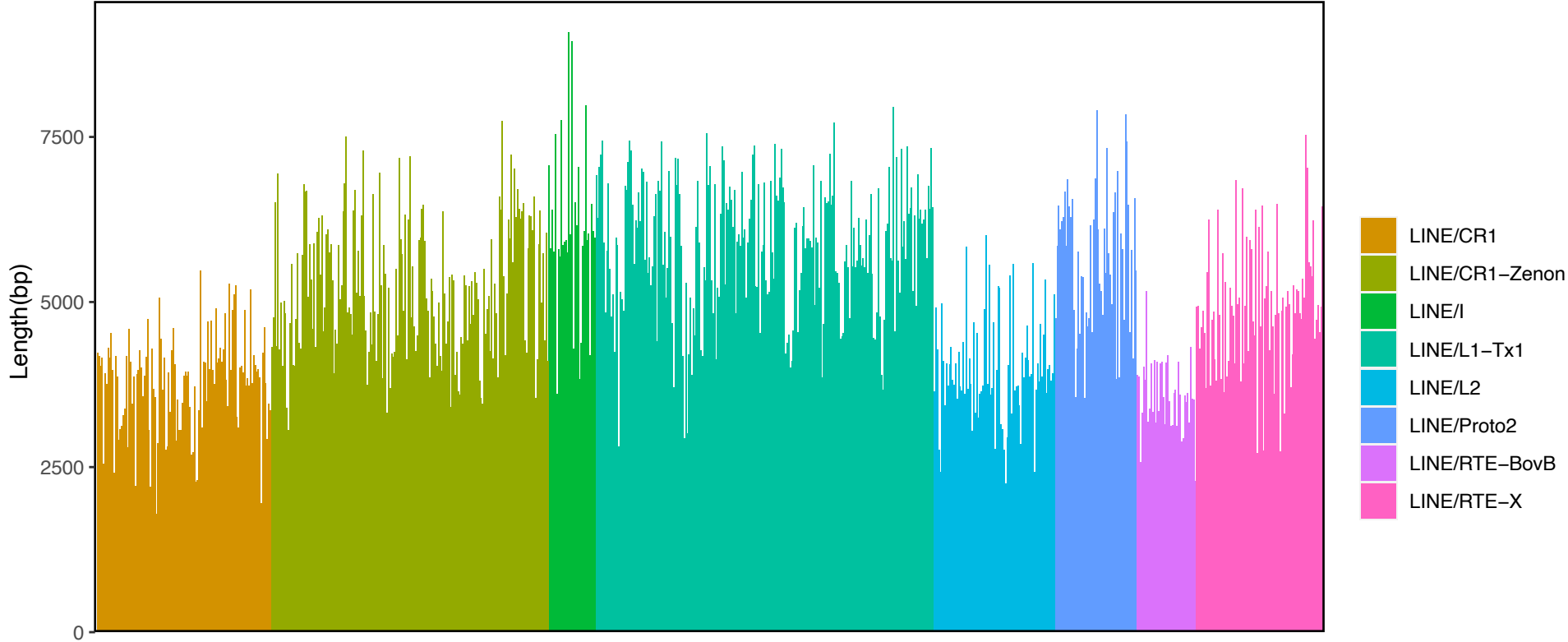

Supplement: Supplementary file 21 — Additional file 21: Fig. S15. Length distribution of manually curated LINE families. Each bar represents an element and colors denote different LINE superfamilies/types. See “Manual curation of LINEs, SINEs, and DDE/D-related transposons” section. [file 12915_2023_1632_MOESM21_ESM.pdf]

Figure S16

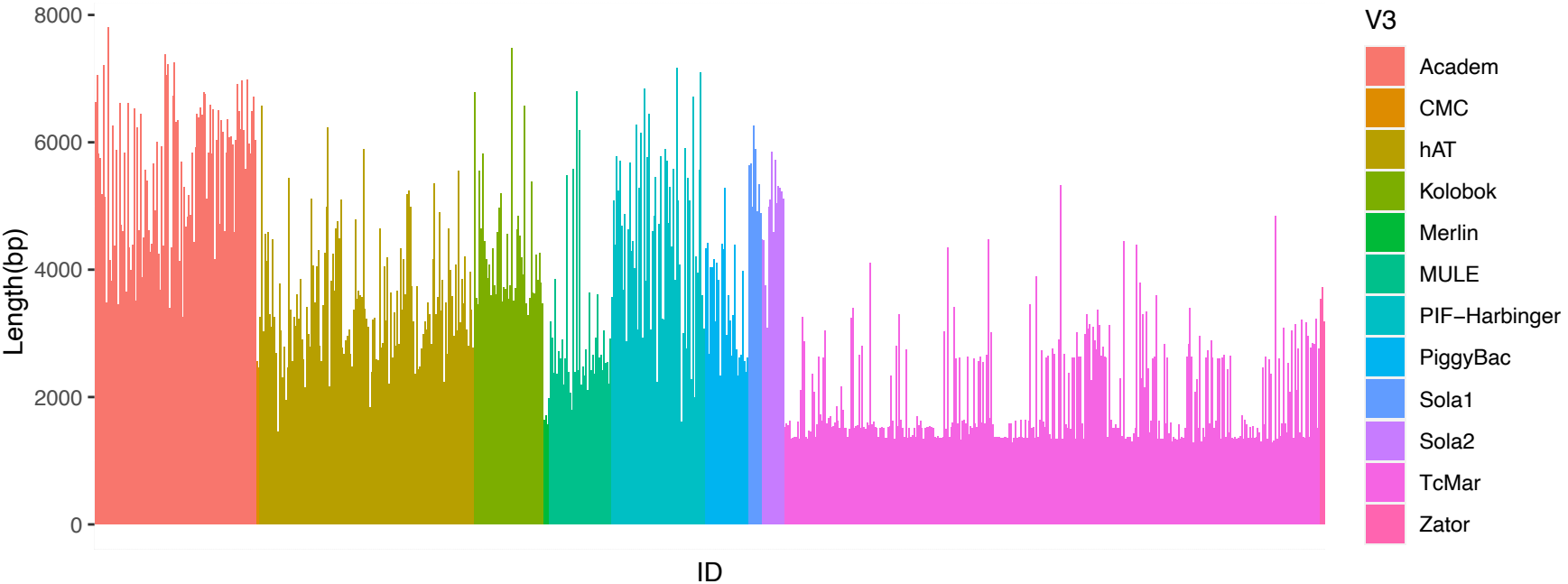

Supplement: Supplementary file 22 — Additional file 22: Fig. S16. Length distribution of manually curated DDE/D transposon families. Each bar represents an element and colors denote different superfamilies. See “Manual curation of LINEs, SINEs, and DDE/D-related transposons” section. [file 12915_2023_1632_MOESM22_ESM.pdf]

Figure S17

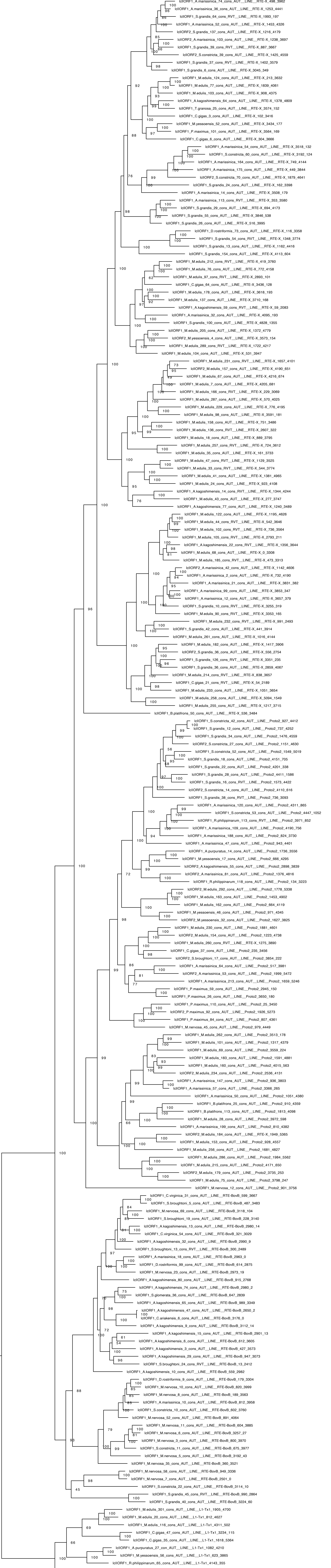

Supplement: Supplementary file 23 — Additional file 23: Fig. S17. Phylogenetic tree of curated bivalves LINEs RTE families. Numbers on nodes represent UltraFast Bootstrap values. See “Genome annotation of LINEs and SINEs using manually curated libraries and phylogenetic inference of curated LINE families” section. [file 12915_2023_1632_MOESM23_ESM.pdf]

Figure S18

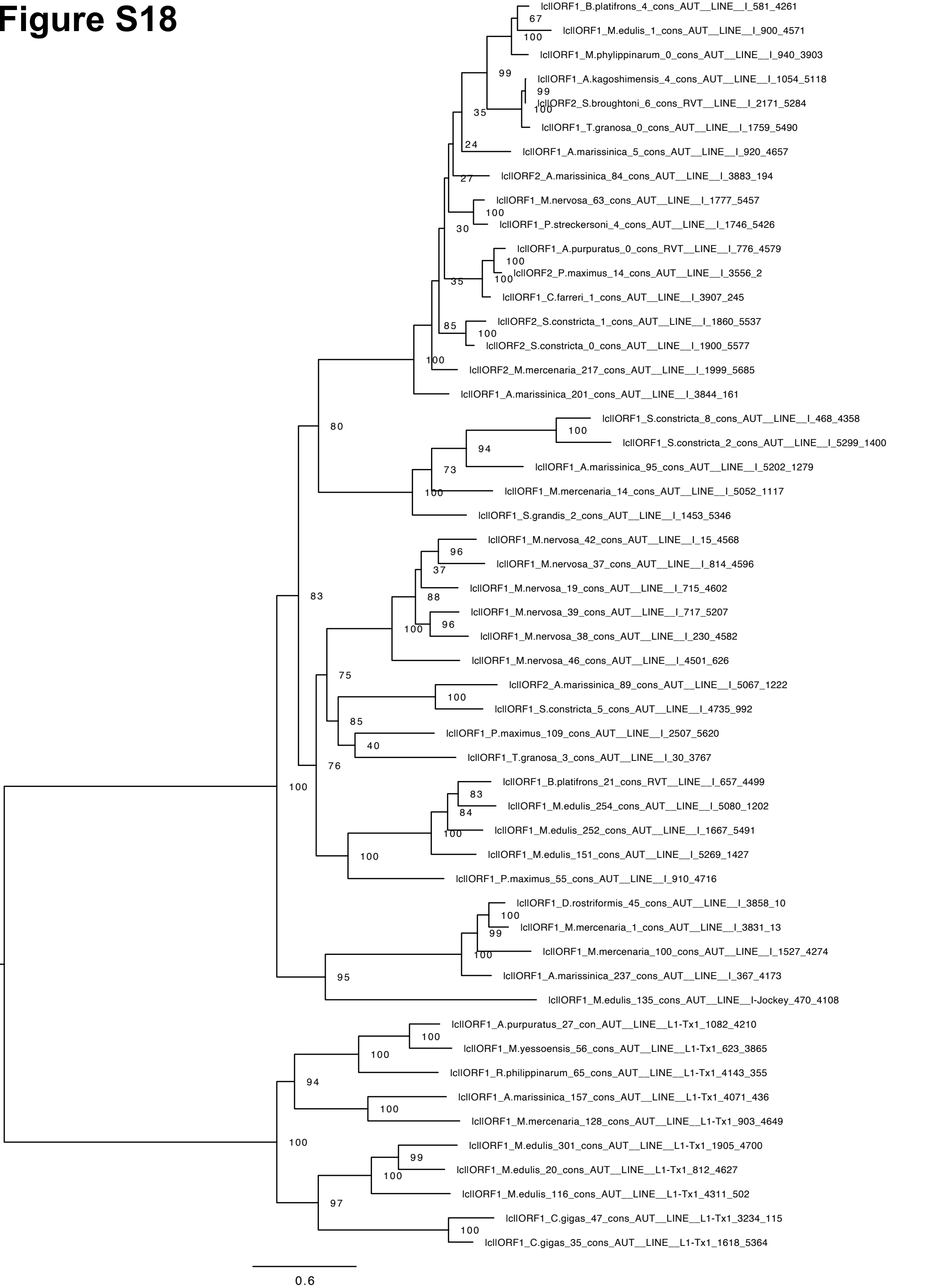

Supplement: Supplementary file 24 — Additional file 24: Fig. S18. Phylogenetic tree of curated bivalves LINEs I families. Numbers on nodes represent UltraFast Bootstrap values. See “Genome annotation of LINEs and SINEs using manually curated libraries and phylogenetic inference of curated LINE families” section. [file 12915_2023_1632_MOESM24_ESM.pdf]

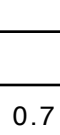

Supplement: Supplementary file 25 — Additional file 25: Fig. S19. Phylogenetic tree of curated bivalves LINEs L1 families. Numbers on nodes represent UltraFast Bootstrap values. See “Genome annotation of LINEs and SINEs using manually curated libraries and phylogenetic inference of curated LINE families” section. [file 12915_2023_1632_MOESM25_ESM.pdf]

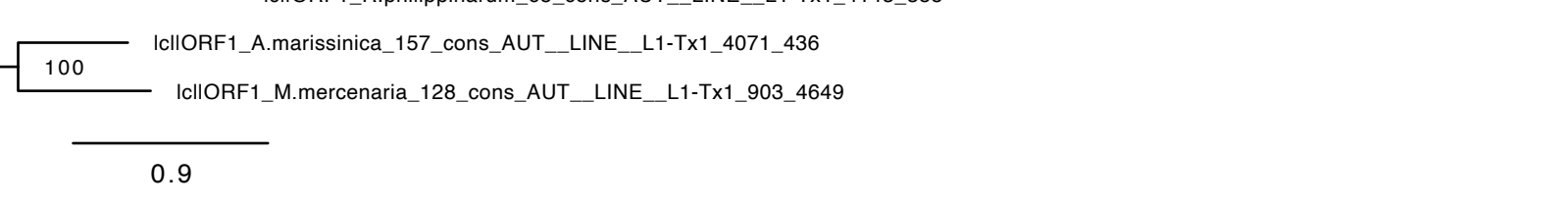

Supplement: Supplementary file 26 — Additional file 26: Fig. S20. Phylogenetic tree of curated bivalves LINEs Jockey families. Numbers on nodes represent UltraFast Bootstrap values. See “Genome annotation of LINEs and SINEs using manually curated libraries and phylogenetic inference of curated LINE families” section. [file 12915_2023_1632_MOESM26_ESM.pdf]
