# Supplementary material for: Multiple and diversified transposon lineages contribute to early and recent bivalve genome evolution
Source: BMC Biol. 2023 Jun 26;21:145. doi: 10.1186/s12915-023-01632-z (PMC10294476; doi:10.1186/s12915-023-01632-z)
Supplement: Supplementary file 3 — Additional file 3: Table S2. Details about the number of de-novo sequences mined from software and used for automatic construction of species-specific repeats libraries (see “Mining and annotation of interspersed repeats” section). [file 12915_2023_1632_MOESM3_ESM.docx]

**Tab. S2:** Details about the number of de-novo sequences mined from software and used for automatic construction of species-specific repeats libraries (See Material and Methods section 5.2).

| **Species** | **RepeatModeler2** | **MITE_tracker** | **HelitronScanner** | **Cleaned Library** |
| --- | --- | --- | --- | --- |
| *Helobdella robusta* | 1,206 | 32 | 0 | 407 |
| *Dinophilus gyrociliatus* | 233 | 14 | 0 | 92 |
| *Capitella teleta* | 2,645 | 238 | 1 | 1,541 |
| *Acanthopleura granulata* | 1,901 | 360 | 0 | 720 |
| *Octopus sinensis* | 3,033 | 2,982 | 9 | 1,735 |
| *Octopus bimaculoides* | 3,138 | 1,736 | 4 | 1,755 |
| *Lottia gigante* | 1,694 | 230 | 0 | 570 |
| *Crysomallon squamiferum* | 1,430 | 90 | 0 | 166 |
| *Pomacea canaliculata* | 1,963 | 285 | 0 | 581 |
| *Biomphalaria glabrata* | 2,426 | 1,557 | 0 | 1,503 |
| *Acanthina immaculata* | 1,960 | 484 | 0 | 809 |
| *Solen grandis* | 3,575 | 3,507 | 39 | 2,157 |
| *Sinonovacula constricta* | 2,202 | 995 | 6 | 1,350 |
| *Dreissena rostriformis* | 3,040 | 3,847 | 8 | 1,975 |
| *Potamilus streckersoni* | 2,441 | 744 | 1 | 1,036 |
| *Megalonaias nervosa* | 2,966 | 1,510 | 0 | 1,442 |
| *Archivesica marissinica* | 2,598 | 5,107 | 44 | 1,795 |
| *Cyclina sinensis* | 2,733 | 929 | 0 | 1,450 |
| *Mercenaria mercenaria* | 4,091 | 4,084 | 6 | 2,864 |
| *Ruditapes philippinarum* | 3,197 | 594 | 42 | 2,332 |
| *Andara kagoshimensis* | 2,775 | 3,216 | 25 | 1,784 |
| *Scapharca broughtonii* | 2,754 | 2,944 | 1 | 1,676 |
| *Tegillarca granosa* | 2,138 | 1,097 | 19 | 1,146 |
| *Myzuhopecten yessoensis* | 2,606 | 1,811 | 0 | 1,519 |
| *Chlamys farreri* | 2,740 | 1,356 | 1 | 1,538 |
| *Pecten maximus* | 2,654 | 846 | 0 | 1,491 |
| *Argopecten irradians concentricus* | 2,910 | 2,030 | 0 | 1,882 |
| *Argopecten purpuratus* | 2,627 | 2,098 | 0 | 1,710 |
| *Pinctada fucata* | 3,366 | 2,519 | 2 | 2,217 |
| *Saccostrea glomerata* | 3,521 | 2,131 | 1 | 2,482 |
| *Crassostrea virginica* | 2,868 | 1,237 | 0 | 1,527 |
| *Crassostrea ariakensis* | 2,971 | 1,794 | 1 | 1,679 |
| *Crassostrea gigas* | 3,063 | 1,597 | 0 | 1,592 |
| *Mytilus coruscus* | 3,788 | 2,664 | 0 | 2,526 |
| *Mytilus edulis* | 3,797 | 3,220 | 28 | 2,702 |
| *Limnoperna fortunei* | 2,642 | 983 | 6 | 1,648 |
| *Modiolus philippinarum* | 4,324 | 7,071 | 4 | 3,736 |
| *Bathymodiolus platifrons* | 4,347 | 2,979 | 1 | 3,373 |
