# Supplementary material for: Multiple and diversified transposon lineages contribute to early and recent bivalve genome evolution
Source: BMC Biol. 2023 Jun 26;21:145. doi: 10.1186/s12915-023-01632-z (PMC10294476; doi:10.1186/s12915-023-01632-z)
Supplement: Supplementary file 4 — Additional file 4: Table S3. Transposable element genomic content of each transposon class using species-specific automatically generated TE sequence libraries (see “Mining and annotation of interspersed repeats” section). [file 12915_2023_1632_MOESM4_ESM.docx]

**Tab. S3:** Transposable element genomic content of each transposon class using species-specific automatically generated libraries (See Material and Methods section 5.2).

Species Class % genome masked

A.granulata LTR 0.640659916154949

A.granulata Unknown 8.09840710573581

A.granulata MITE 2.69077613232627

A.granulata DNA 1.18273490393162

A.granulata RC 0.358924722493238

A.granulata SINE 3.93486146363796

A.granulata LINE 1.14522440984682

A.i.concentricus LTR 0.844328278973033

A.i.concentricus LINE 2.75537538090985

A.i.concentricus DNA 2.53193022216097

A.i.concentricus RC 3.11938299188601

A.i.concentricus Unknown 12.4842828279804

A.i.concentricus MITE 9.0770142406302

A.i.concentricus SINE 0.862671214265486

A.immaculata LTR 0.433130010853187

A.immaculata SINE 0.0367441271926965

A.immaculata MITE 6.27083795231979

A.immaculata DNA 4.73567810919043

A.immaculata Unknown 5.43692857716181

A.immaculata RC 0.22413883107998

A.immaculata LINE 24.7797041110103

A.kagoshimensis MITE 6.42117114429528

A.kagoshimensis LINE 6.45912292159699

A.kagoshimensis DNA 5.5611126139914

A.kagoshimensis Unknown 10.8584060733432

A.kagoshimensis LTR 1.40011384923454

A.kagoshimensis RC 4.43679645695323

A.kagoshimensis SINE 5.35438566442369

A.marissinica MITE 14.5983660303479

A.marissinica Unknown 8.00237091456887

A.marissinica SINE 3.05355583466292

A.marissinica LINE 5.068667739608

A.marissinica LTR 1.20585737624975

A.marissinica DNA 3.26272627550336

A.marissinica RC 0.379115010594951

A.purpuratus LTR 0.562928717339136

A.purpuratus DNA 2.26413711175394

A.purpuratus LINE 2.39199235935374

A.purpuratus SINE 1.29786163033155

A.purpuratus RC 2.20177644495815

A.purpuratus MITE 9.09183609247276

A.purpuratus Unknown 9.98033382726857

B.glabrata DNA 3.50307849166989

B.glabrata SINE 0.0296502167508138

B.glabrata Unknown 13.9697323543998

B.glabrata LTR 0.87010100655211

B.glabrata RC 3.56177065851601

B.glabrata LINE 10.6817262141498

B.glabrata MITE 6.17973222897077

B.platifrons DNA 4.11597964381163

B.platifrons RC 3.41214544067715

B.platifrons LTR 1.25775316927925

B.platifrons LINE 5.62602625061985

B.platifrons MITE 6.85669937692548

B.platifrons Unknown 18.7037189254911

B.platifrons SINE 6.22702319895405

C.ariakensis LINE 2.84695764706319

C.ariakensis DNA 14.3672536989628

C.ariakensis MITE 5.77988320122633

C.ariakensis Unknown 3.72621323919029

C.ariakensis RC 14.8457041163975

C.ariakensis SINE 0.153388101701154

C.ariakensis LTR 1.92606983799261

C.farreri DNA 1.33236641415706

C.farreri LINE 2.36194192561845

C.farreri RC 2.7103476122865

C.farreri MITE 7.46848456015607

C.farreri SINE 1.00809035102616

C.farreri LTR 0.384959828723053

C.farreri Unknown 7.16179371125094

C.gigas MITE 6.00249290039496

C.gigas SINE 0.0474243122640857

C.gigas RC 12.4853878668925

C.gigas LTR 1.93057834581324

C.gigas LINE 2.28254738649317

C.gigas DNA 14.1631210784863

C.gigas Unknown 3.50685668926047

C.sinensis Unknown 10.1589025311947

C.sinensis DNA 3.23068648769506

C.sinensis MITE 4.47633106553755

C.sinensis SINE 1.22769712850167

C.sinensis LTR 1.04298623225558

C.sinensis RC 10.8229023502664

C.sinensis LINE 1.75483340405576

C.squamiferum Unknown 1.38222921833381

C.squamiferum LINE 1.29163673236377

C.squamiferum RC 0.0160485800788

C.squamiferum MITE 0.427587211341659

C.squamiferum DNA 3.97350954913994

C.squamiferum LTR 2.5954514540215

C.teleta MITE 3.05354027917302

C.teleta RC 0.00849397729032901

C.teleta LTR 2.62357382253714

C.teleta LINE 6.25737525906196

C.teleta SINE 0.845583255427618

C.teleta Unknown 7.8665235363433

C.teleta DNA 5.21628110348722

C.virginica LTR 1.69337425865852

C.virginica LINE 1.61393387195518

C.virginica DNA 8.39272327161864

C.virginica RC 9.61016452921461

C.virginica MITE 5.62510128645289

C.virginica SINE 0.00888131258854368

C.virginica Unknown 6.10601222714929

D.gyrociliatus LTR 1.46714174551359

D.gyrociliatus MITE 0.581109126516605

D.gyrociliatus Unknown 1.24805440808593

D.gyrociliatus LINE 0.0672385782064565

D.gyrociliatus DNA 1.91458889207186

D.rostriformis RC 2.3151058103041

D.rostriformis Unknown 10.5823374549799

D.rostriformis LINE 3.31011818382682

D.rostriformis DNA 1.93535278687331

D.rostriformis LTR 1.02640118327612

D.rostriformis MITE 8.26664388932791

D.rostriformis SINE 0.414123863948635

H.robusta LINE 7.51122174989601

H.robusta MITE 0.4549810647993

H.robusta Unknown 1.83515987126122

H.robusta LTR 1.92318237620734

H.robusta DNA 6.25122205978295

L.fortunei Unknown 12.805005455256

L.fortunei LTR 0.773637957933607

L.fortunei RC 3.79424759533367

L.fortunei SINE 2.28697781423494

L.fortunei DNA 2.97596093507115

L.fortunei MITE 3.96820868280699

L.fortunei LINE 5.62356325590404

L.gigantea SINE 1.85641801897822

L.gigantea LTR 0.203063279658779

L.gigantea Unknown 5.92373024839208

L.gigantea LINE 0.895945540419129

L.gigantea DNA 3.27819615906584

L.gigantea RC 0.698879106406745

M.coruscus SINE 0.95668213140782

M.coruscus DNA 4.10878875906935

M.coruscus MITE 6.94854936120602

M.coruscus Unknown 20.0713157388761

M.coruscus LINE 10.8404445316129

M.coruscus LTR 2.15573026603721

M.coruscus RC 4.12557362820091

M.edulis DNA 3.26810788065409

M.edulis RC 3.82263604650232

M.edulis LINE 9.17772017422381

M.edulis Unknown 19.2812175823921

M.edulis SINE 0.805249646772649

M.edulis LTR 3.20869940631906

M.edulis MITE 8.19138562273354

M.mercenaria LINE 3.41233658914872

M.mercenaria Unknown 16.2573880437828

M.mercenaria LTR 2.74208091217912

M.mercenaria SINE 2.19310959314128

M.mercenaria MITE 10.099137909058

M.mercenaria RC 11.0962037975613

M.mercenaria DNA 5.01061640177892

M.nervosa LTR 6.66106120245081

M.nervosa DNA 18.391162750345

M.nervosa SINE 0.926441453466806

M.nervosa Unknown 13.6270820753434

M.nervosa MITE 3.63049356591277

M.nervosa LINE 4.75343480786718

M.phylippinarum DNA 4.52980334543074

M.phylippinarum LINE 8.5674268303119

M.phylippinarum RC 10.7684218150225

M.phylippinarum SINE 0.0622065755988345

M.phylippinarum MITE 16.7354695075285

M.phylippinarum Unknown 16.2421169432329

M.phylippinarum LTR 1.64999150871677

M.yessoensis MITE 3.52989815798143

M.yessoensis RC 3.59527446728392

M.yessoensis LINE 3.50657387172805

M.yessoensis LTR 0.771881503852949

M.yessoensis DNA 1.6492513622833

M.yessoensis SINE 0.597402987122673

M.yessoensis Unknown 7.91297452295305

O.bimaculoides Unknown 9.80222562713501

O.bimaculoides LTR 1.78349204824814

O.bimaculoides LINE 9.7266499074325

O.bimaculoides DNA 9.92130194045213

O.bimaculoides SINE 2.69460812937901

O.bimaculoides RC 0.305937059277192

O.bimaculoides MITE 6.66234476870397

O.sinensis RC 0.33358938106136

O.sinensis SINE 1.28840595386495

O.sinensis MITE 11.8518351535625

O.sinensis DNA 14.7363076224346

O.sinensis Unknown 11.4210748127598

O.sinensis LINE 16.6828052035763

O.sinensis LTR 1.07801888443377

P.canaliculata LINE 3.81223880725598

P.canaliculata DNA 1.68005709674089

P.canaliculata SINE 0.131812408127648

P.canaliculata MITE 1.3451417797467

P.canaliculata LTR 0.464122306683904

P.canaliculata Unknown 4.12620354292196

P.fucata MITE 10.5335811190384

P.fucata LINE 7.65743221743945

P.fucata Unknown 13.5850686617618

P.fucata LTR 0.756206453484661

P.fucata RC 5.96877493220955

P.fucata SINE 0.21010003726408

P.fucata DNA 5.30549795650273

P.maximus RC 4.93669445035696

P.maximus DNA 2.86630166473699

P.maximus MITE 3.79197442533716

P.maximus SINE 0.322539521771676

P.maximus LTR 1.33378219877724

P.maximus LINE 2.62724386740566

P.maximus Unknown 13.5850686617618

P.streckersoni SINE 1.52488230168523

P.streckersoni DNA 15.4642036136419

P.streckersoni LTR 2.66158090122471

P.streckersoni MITE 3.47952098800825

P.streckersoni Unknown 11.5139711448206

P.streckersoni RC 0.057256943541308

R.philippinarum Unknown 14.7659725152652

R.philippinarum SINE 1.25446059911696

R.philippinarum MITE 9.22443648831968

R.philippinarum LTR 1.83590433735259

R.philippinarum LINE 2.94622234228432

R.philippinarum DNA 3.53118855524124

R.philippinarum RC 6.14901140940591

S.broughtonii LTR 0.494643452511471

S.broughtonii SINE 5.58077280470772

S.broughtonii MITE 6.69966857420843

S.broughtonii DNA 5.00329653170949

S.broughtonii RC 4.45769261049181

S.broughtonii Unknown 10.3077672979623

S.broughtonii LINE 7.55493710791791

S.constricta LTR 1.22411981467075

S.constricta MITE 5.06434448555706

S.constricta SINE 3.1450532589976

S.constricta DNA 3.46323681785627

S.constricta RC 1.35583192595906

S.constricta Unknown 12.2470522919315

S.constricta LINE 3.07278591732062

S.glomerata LTR 1.40030774710538

S.glomerata SINE 0.171153567403316

S.glomerata DNA 8.63386576685821

S.glomerata MITE 7.40211324707039

S.glomerata RC 10.2999429240684

S.glomerata Unknown 8.83402372735953

S.glomerata LINE 5.59264585859725

S.grandis RC 7.73199131145613

S.grandis LTR 1.85935121530911

S.grandis Unknown 16.1175339575861

S.grandis LINE 3.40664557987496

S.grandis MITE 7.78023276238463

S.grandis SINE 4.66145988723136

S.grandis DNA 4.663981911394

T.granosa SINE 4.99007947133166

T.granosa Unknown 5.52520276164394

T.granosa RC 6.49789760344305

T.granosa MITE 2.55500656173098

T.granosa DNA 5.12369070166856

T.granosa LINE 4.0131347626811

T.granosa LTR 0.843751707636531
