# Supplementary material for: Multiple and diversified transposon lineages contribute to early and recent bivalve genome evolution
Source: BMC Biol. 2023 Jun 26;21:145. doi: 10.1186/s12915-023-01632-z (PMC10294476; doi:10.1186/s12915-023-01632-z)
Supplement: Supplementary file 12 — Additional file 12: Table S6. Results of Maximum likelihood (ML) topology test between constrained and unconstrained tree searches (see “Tree-based classification of ORF-containing LINE elements” section). Tree with the highest log likelihood is highlighted in bold. FullNJ=ML tree obtained with a full constrain on the topology recovered by Neighbour-Joining; SupFAM=ML tree constrained only on the superfamilies relationships obtained by Neighbour-Joining. Plus signs denote accepted topologies by the respective topology test. [file 12915_2023_1632_MOESM12_ESM.docx]

**Tab. S6:** Results of Maximum likelihood (ML) topology test between constrained and unconstrained tree searches (See Material and Methods section 5.4). Tree with the highest log likelihood is highlighted in bold. **FullNJ=**ML tree obtained with a full constrain on the topology recovered by Neighbour-Joining; **SupFAM=**ML tree constrained only on the superfamilies relationships obtained by Neighbour-Joining. Plus signs denote accepted topologies by the respective topology test.

| **Tree** | **logL** | **deltaL** | **bp-RELL** | **p-KH** | **p-SH** | **c-ELW** | **p-AU** |
| --- | --- | --- | --- | --- | --- | --- | --- |
| **Uncostrained #1** | -672720.5759 | 183.57 | 0.009 - | 0.08 + | 0.676 + | 0.00841 - | 0.108 + |
| **Uncostrained #2** | -672775.2211 | 238.22 | 0.001 - | 0.026 - | 0.602 + | 0.00101 - | 0.0653 + |
| **Uncostrained #3** | -672549.6755 | 12.67 | 0.222 + | 0.456 + | 0.936 + | 0.222 + | 0.591 + |
| **Uncostrained #4** | -672895.6293 | 358.62 | 0.008 - | 0.03 - | 0.508 + | 0.00795 - | 0.0354 - |
| **Uncostrained #5** | -672649.7246 | 112.72 | 0.043 + | 0.206 + | 0.831 + | 0.0422 + | 0.242 + |
| **FullNJ** | -703136.8519 | 30600 | 0 - | 0 - | 0 - | 0 - | 3.84e-71 - |
| **SupFAM #1** | -672594.0508 | 57.045 | 0.105 + | 0.334 + | 0.893 + | 0.107 + | 0.335 + |
| **SupFAM #2** | **-672537.0059** | **0** | **0.238 +** | **0.544 +** | **1 +** | **0.237 +** | **0.643 +** |
| **SupFAM #3** | -672550.7819 | 13.776 | 0.225 + | 0.467 + | 0.934 + | 0.226 + | 0.553 + |
| **SupFAM #4** | -672577.263 | 40.257 | 0.119 + | 0.378 + | 0.919 + | 0.118 + | 0.43 + |
| **SupFAM #5** | -672649.5896 | 112.58 | 0.03 - | 0.191 + | 0.812 + | 0.0306 - | 0.243 + |
