# Supplementary material for: Multiple and diversified transposon lineages contribute to early and recent bivalve genome evolution
Source: BMC Biol. 2023 Jun 26;21:145. doi: 10.1186/s12915-023-01632-z (PMC10294476; doi:10.1186/s12915-023-01632-z)
Supplement: Supplementary file 28 — Additional file 28: Table S8. Conserved Domain Database identifier used to search for Reverse Transcriptase and Endonuclease signatures in extracted open reading frames. [file 12915_2023_1632_MOESM28_ESM.docx]

**Tab. S8:** Conserved Domain Database identifier used to search for Reverse Transcriptase and Endonuclease signatures in extracted open reading frames.

| TE Subclass/Superfamily | Domain | CDD RPSSMs | CDD Profile name |
| --- | --- | --- | --- |
| LINE | RVT | CDD:238185 | RT_like |
|  |  | CDD:238827 | RT_nLTR_like |
|  |  | CDD:395031 | RVT_1 |
|  |  | CDD:238828 | RT_G2_intron |
|  |  | CDD: 400190 | RVT_2 |
|  | EN | CDD: 412407 | EEP Superfamily |
|  |  | CDD: 197318 | EEP-2 |
|  |  | CDD:397447 | Exo_endo_phos |
|  |  | CDD:405252 | Exo_endo_phos_2 |
|  |  | CDD: 197310 | L1-EN |
|  |  | CDD: 197311 | R1-I-EN |
