# Supplementary material for: Multiple and diversified transposon lineages contribute to early and recent bivalve genome evolution
Source: BMC Biol. 2023 Jun 26;21:145. doi: 10.1186/s12915-023-01632-z (PMC10294476; doi:10.1186/s12915-023-01632-z)

Figure S8

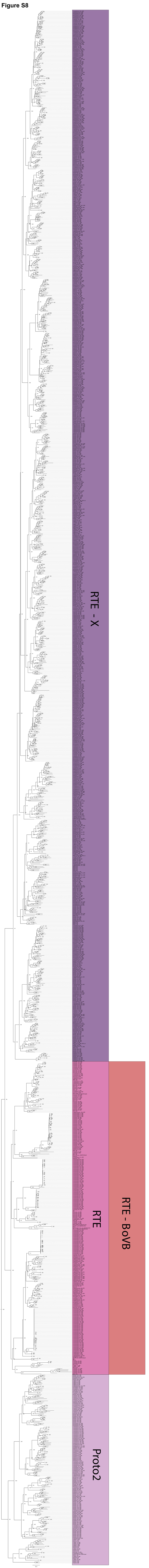

Supplement: Supplementary file 14 — Additional file 14: Fig. S8. RTE superfamily subtree extracted from the SupFAM tree #2. Numbers on nodes represent UltraFast Bootstrap values. The outer-left annotation refers to the classification scheme proposed by RepBase and based on [29] while the outer-right based on the RepeatMasker “type” and obtained through blastp against the RepeatPep library. See “Tree-based classification of ORF-containing LINE elements” section. [file 12915_2023_1632_MOESM14_ESM.pdf]
